# Phospho‐Proteomics Identifies D‐Group MAP Kinases as Substrates of the *Arabidopsis* Tyrosine Phosphatase RLPH2

**DOI:** 10.1002/pld3.70137

**Published:** 2026-01-20

**Authors:** Anne‐Marie Labandera, Ryan Toth, Sierra Mitchell, Jayde J. Johnson, Brooklyn Kurucz, Juliette Puyaubert, Emmanuel Baudouin, R. Glen Uhrig, Greg B. Moorhead

**Affiliations:** ^1^ Department of Biological Sciences University of Calgary Calgary Canada; ^2^ INRAE, ONF, UMR BioForA Orléans France; ^3^ Sorbonne Université, CNRS, Inserm, Development, Adaptation and Ageing, Dev2A Paris France; ^4^ Sorbonne Université, CNRS, Inserm, Institut de Biologie Paris‐Seine, IBPS Paris France; ^5^ Department of Biological Sciences University of Alberta Edmonton Alberta Canada; ^6^ Department of Biochemistry University of Alberta Edmonton Alberta Canada

**Keywords:** *Arabidopsis thaliana*, mitogen‐activated protein kinase, phospho‐proteomics, protein kinase activation loop, protein phosphatase one (PP1), seed dormancy, tyrosine phosphatase

## Abstract

Despite being one of the few bona fide plant tyrosine phosphatases, the *Arabidopsis thaliana* *Rhizobiales*‐like phosphatase 2 (RLPH2) has no known substrates. Utilizing phospho‐proteomics, we identified the activation loop phospho‐tyrosine of several *A. thaliana* D‐group mitogen‐activated protein kinases (MPKs) as potential RLPH2 substrates. All *Arabidopsis* D‐group MPKs possess a TDY activation loop phosphorylation motif, whereas other MPKs (Groups A, B, and C) contain a TEY motif. Our findings reveal that RLPH2 has a strong preference for aspartate (D) in the TXY motif, providing specificity for RLPH2 to exclusively target and dephosphorylate the D‐group MPKs. Additionally, D‐group MPKs contain a unique activation loop insertion that conforms to a protein phosphatase one (PP1) binding motif, with findings presented here confirming *Arabidopsis* PP1 phosphatases dock at this site. Intriguingly, only D‐group MPKs among all identified *Arabidopsis* protein kinases possess this PP1 recruiting motif. Using multiple RLPH2‐deficient plant lines, we demonstrate that RLPH2 represses seed dormancy release. Overall, this work highlights the power of phospho‐proteomics in identifying substrates of this novel plant tyrosine phosphatase while also revealing new complexities in the interactions between MPK activation loops and multiple phospho‐mediated cell signaling events.

**Abbreviations**
ABA: abscisic acid
CD: common docking
GA: gibberellic acid
KIM: kinase interacting motif
MAP kinases: mitogen‐activated protein kinases
MAPKK: MAPK kinase
MPKs: mitogen‐activated protein kinases
PP1: protein phosphatase one
pS: phospho‐serine
pT: phospho‐threonine
pY: phospho‐tyrosine
RLPH2: *Rhizobiales*‐like phosphatase 2
SLiM: short linear motif

## 1 Introduction

Protein phosphorylation machinery, composed of protein kinases and phosphatases, is highly conserved across eukaryotic organisms with protein phosphorylation recognized as a fundamental mechanism of controlling protein function. Proteins are primarily phosphorylated on the hydroxy amino acids serine (pS), threonine (pT), and tyrosine (pY) with eukaryotic phospho‐proteomes being approximately 86%, 12%, and 2% on each of these residues, respectively (Kerk et al. 2023; Brautigan and Shenolikar 2018), including plants (Sugiyama et al. 2008). The protein phosphatases that remove the phosphate from these protein sidechains are highly conserved across eukaryotes and are divided into four groups based primarily on primary amino acid sequence defining catalytic motifs and domains. These four groups include the protein tyrosine phosphatases (PTP), the aspartate‐dependent phosphatases, and the phosphoprotein phosphatases (PPP) composed of PP1, PP2A, PP2B, PP4‐PP7‐like, and the Mg^2+^‐ or Mn^2+^‐dependent protein phosphatase (PPM) family (Brautigan and Shenolikar 2018; Uhrig et al. 2013; Kerk et al. 2021). Although the tyrosine phosphatases, based on their characterization in humans, display substrate specificity because of differing active site architecture, the serine/threonine specific enzymes from the PPP family are thought to achieve much of their specificity from associated regulatory subunits (Brautigan and Shenolikar 2018; Uhrig et al. 2013). This is best characterized in the Type 1 protein phosphatases (PP1 or TOPP in plants), the majority of which dock the PP1 catalytic subunit via a short linear motif (SLiM) designated RVXF (Kerk et al. 2023; Brautigan and Shenolikar 2018; Kerk et al. 2008).

Like humans, plants maintain all four conserved protein phosphatase families, with the exception that plants have very few “mammalian‐type” protein tyrosine phosphatases, despite possessing tyrosine phosphorylation levels that parallel other eukaryotes (Sugiyama et al. 2008; Labandera et al. 2018; Uhrig et al. 2016). Recently, we characterized the *Arabidopsis thaliana* (At) cytosolic protein phosphatase RLPH2, which based on its primary amino acid sequence is a PPP family serine/threonine protein phosphatase (like PP1) but possesses a clear preference for phospho‐tyrosine peptides as substrates when compared to phosphoserine/threonine peptides (Uhrig et al. 2016). RLPH2 selectively dephosphorylated the pY and not the pT of a human ERK1/2 activation loop peptide, and the presence of pT increased activity against the pY site (Labandera et al. 2018). The mechanism underlying this specificity became clear when we solved the structure of RLPH2 (Labandera et al. 2018). Uniquely, the active site of RLPH2 is deeper compared to pS/pT specific phosphatases (e.g., PP1 or PP2A), allowing the larger pY sidechain to reach the base of the pocket to be dephosphorylated, whereas pS/pT were modeled to be too short to allow for efficient dephosphorylation (Uhrig et al. 2013). Crystallization with the phosphate mimetic tungstate revealed that in addition to the active site, a basic pocket adjacent to the active site also bound tungstate, suggesting that this was an additional site for binding a phospho‐residue of substrates, much like the human dual specificity phosphatase VHR/DUSP3 (Labandera et al. 2018; Schumacher et al. 2002). Further modeling demonstrated that when a substrate pY peptide bound the RLPH2 active site, a pT two residues away was perfectly positioned to dock in the basic pocket. This configuration suggests that the basic pocket likely acts to recruit substrates to RLPH2 that are phosphorylated on threonine, facilitating dephosphorylation on a pY two amino acids C‐terminal to pT, thus providing additional substrate specificity to the enzyme. Further analysis of plant phospho‐proteome databases (data extracted from Plant PTM Viewer 2.0: /https://www.psb.ugent.be/webtools/ptm‐viewer/ptm.php) has uncovered multiple examples of pTxpY within protein sequences (see Table S1) and these could represent potential RLPH2 substrates.

To discover RLPH2 substrates, we undertook a phospho‐proteomics approach. Knowing that biochemically RLPH2 is a tyrosine phosphatase, we isolated phospho‐tyrosine‐containing peptides from the rosettes of *Arabidopsis* wild‐type (WT) Nössen and two *rlph2* knockout lines. In the absence of RLPH2, proteomic analysis revealed an enrichment of tyrosine phosphorylated peptides derived from the activation loops of a subset of *Arabidopsis* mitogen‐activated protein kinases (MPKs), specifically D‐group MPKs. We then validated RLPH2 to specifically target D‐group MPKs, discriminating between the TDY (D‐group MPKs only) versus TEY (all other MPKs) activation loop motifs. Additionally, we uniquely find that D‐group MPKs dock protein phosphatase one (PP1 or TOPP) through a highly conserved PP1 binding RVXF SLiM. Further examination of multiple *rlph2* plant lines indicates that RLPH2 is a negative regulator of germination in both an abscisic acid (ABA)– and gibberellic acid (GA)–dependent manner. With limited biological roles attributed to D‐group MPKs to date, these findings offer critical new molecular, biochemical, and biological insights into their roles in plants.

## 2 Results

### 2.1 Phospho‐Proteomics to Uncover *A. thaliana* RLPH2 Substrates

**FIGURE 1:**
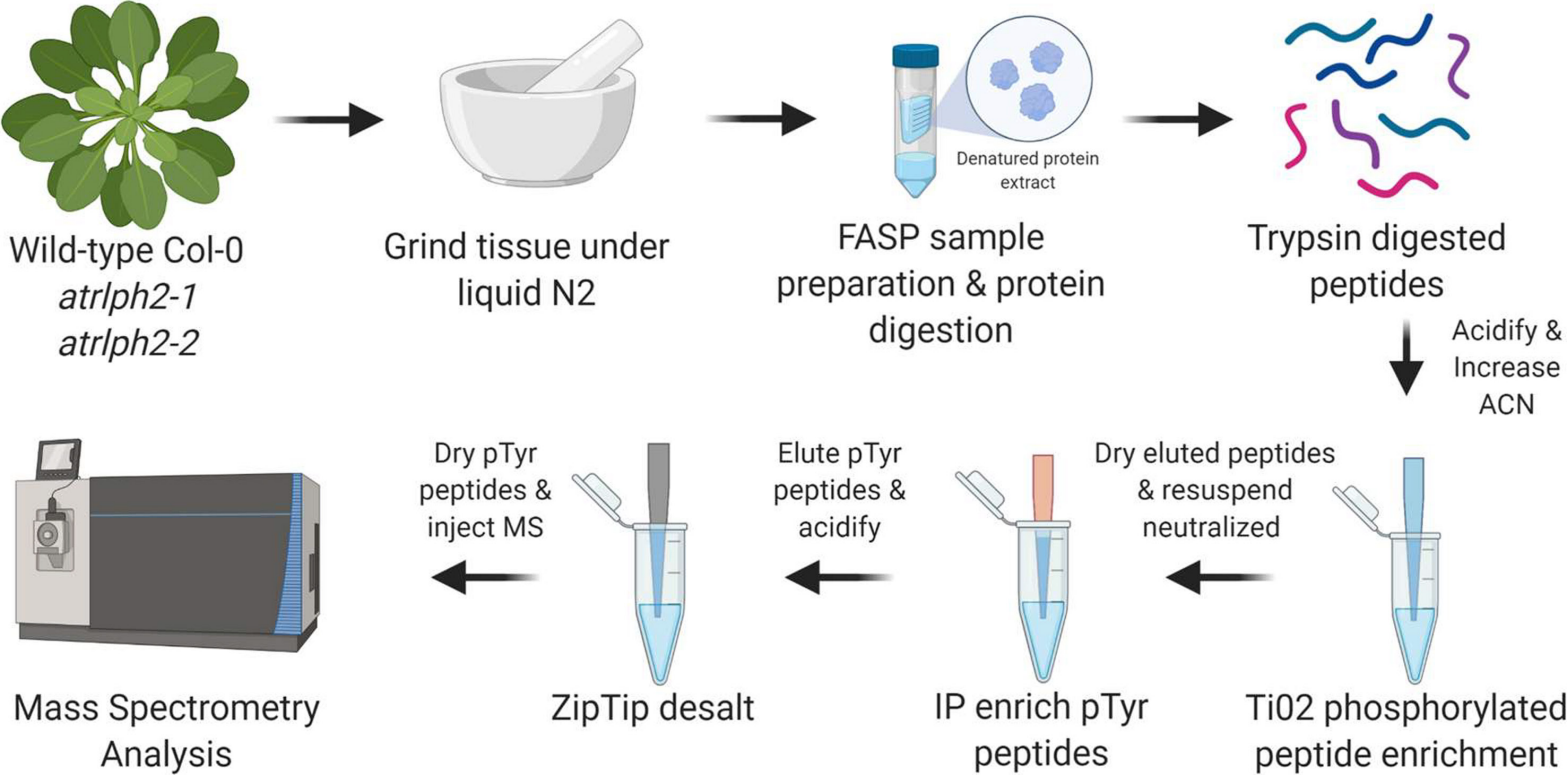
Schematic of the RLPH2 phospho‐tyrosine (pY) substrate enrichment pipeline. Image generated with BioRender.

To identify substrates of the tyrosine phosphatase RLPH2, we undertook a phospho‐proteomic comparison of WT *A. thaliana* Nössen plants and two independent *rlph2* (*rlph2‐1* and *rlph2‐2*) mutant alleles. Biochemically, RLPH2 behaves as a tyrosine phosphatase (Labandera et al. 2018; Uhrig et al. 2016), making the goal of this comparison to elucidate the endogenous substrates of RLPH2 through the identification of tyrosine phosphorylation events present in *rlph2* versus WT plants. Here, we expect that the loss of RLPH2 should result in enrichment of tyrosine phosphorylated endogenous substrates. To achieve this, we first individually isolated the global phospho‐peptide pool of rosette tissue from WT and both *rlph2* plant lines using TiO_2_. We then further enriched the tyrosine phosphorylated fraction from the global phospho‐peptide pool of each plant line by immunoprecipitation (IP) using anti‐phospho‐tyrosine (pY) specific antibodies (Figure 1). Proteomic analysis of anti‐pY IP eluates revealed tyrosine phosphorylated peptides belonging to the activation loop of three D‐group mitogen activated protein kinases (MPK9, MPK18, and MPK20) being specifically detected in the *rlph2* lines, but absent in WT plants (Table 1). Notably, we found phospho‐tyrosine peptides corresponding to the activation loop of other MPK groups were enriched in both *rlph2* and WT plants, suggesting a D‐group MPK specificity for RLPH2 (Table S2).

**TABLE 1:** RLPH2 preferentially dephosphorylates D‐group MAP kinases (MPKs).

AGI	Description	Phosphorylated peptide
PTM localization probability	MASCOT SCORE	WT_Col‐0	*atrlph2‐1*	*atrlph2‐2*
AT2G42880	ATMPK20, MPK20 | MAP kinase 20	VAFNDTPTTIFWTD **(pY)**VATR	100%	48.35	0/2	1/2	2/2
AT3G18040	ATMPK9 | MAP kinase 9	VSFNDAPSAIFWTD **(pY)**VATR	100%	39.53	0/2	1/2	2/2
AT1G53510	ATMPK18, MPK18 | MAP kinase 18	VAFNDTPTTVFWTD **(pY)**VATR	69%	35.27	0/2	0/2	2/2
AT5G19010	ATMPK16 | MAP kinase 16	VAFNDTPTAIFWTD **(pY)**VATR	99%	48.41	1/2	2/2	2/2
AT2G43790	ATMPK6, MPK6, MAPK6, ATMAPK6 | MAP kinase 6	VTSESDF (oxM)TE **(pY)**VVTR	100%, 100%	96.41	2/2	2/2	2/2
AT3G45640	APK3, MPK3, AAPK3 | MAP kinase 3	PTSENDF (oxM)TE **(pY)**VVTR	100%, 100%	58.72	2/2	1/2	2/2
AT1G10210	ATMPK1, MPK 1 | MAP kinase 1	GQF (oxM)TE (**pY)**VVTR	100%, 100%	46.68	2/2	1/2	2/2

*Note:* *Arabidopsis thaliana* D‐group MAP kinases (MPKs) are exclusively phosphorylated in *rlph2‐1* and *rlph2‐2* relative to other MPKs. Shown are the tabulated results of two complete independent experiments in which phosphorylated‐tyrosine (pY) containing peptides were isolated through a sequential Ti0_2_ enrichment/anti‐pY immunoprecipitation workflow (Figure 1). The complete list of all anti‐pY immunoprecipitated phosphorylated proteins are found in Table S2. Highlighted in bold is the phosphorylated tyrosine residue (pY) identified, whereas identified oxidized methionine residues are denoted (oxM). The number of independent experiments in which each phosphorylated peptide is found (x/2) is shown under the corresponding genotype delimiter.

### 2.2 Sequence Analysis of D‐Group MPKs

*A. thaliana* encodes 20 mitogen‐activated protein kinases (MPKs) that phylogenetically form four distinct clusters designated Groups A–D (Figure S1). Unlike Groups A, B, and C, the D‐group MPKs possess a 60–80‐amino‐acid C‐terminal extension (Ichimura et al. 2002). Alignment of their activation loop, or T‐loops, reveals that the D‐group enzymes are also distinct from the A‐, B‐, and C‐group MPKs in this region (Figure S2). MPKs are thought to require dual phosphorylation on both the T and Y (TXY motif) of the T‐loop for full kinase activation (Zhang et al. 2008). Uniquely, the D‐group enzymes have an aspartate (D) in the activation loop motif (TDY), whereas the other groups have glutamate (E) (TEY). Just N‐terminal to this motif, but still within the activation loop (defined by DFGX_19–25_ APE), the D‐group MPKs have an insert, making this surface exposed loop longer, and uniquely each insert has a conserved putative PP1 docking site (defined as RVXF [Brautigan and Shenolikar 2018; Uhrig et al. 2013]), which is not seen in the A, B, and C groups (Figures S2 and S3). The MPK enzymes, including the *Arabidopsis* A, B, and C groups, have a common docking (CD) domain that has been characterized as a common binding site for the activating MPK kinase (MPKK), the inactivating MAPK phosphatase, and MPK substrates via their KIM (kinase interacting motif) sequence (Ichimura et al. 2002; Tanoue et al. 2002; Bardwell et al. 2009; Peti and Page 2013). The CD domain is absent in the D‐group MPKs, further adding to their uniqueness (Ichimura et al. 2002; Bardwell et al. 2009). This absence is consistent with the notion that D‐group MPKs do not appear to be phosphorylated by an upstream kinase and rather autophosphorylate to activate (Nagy et al. 2015). Consequently, we predict D‐group MPKs have evolved a mechanism for dephosphorylation and inactivation independent of docking MAPK phosphatases via a CD domain (Ichimura et al. 2002; Tanoue et al. 2002). Together, this suggests that D‐group MPKs autophosphorylate to activate and are likely dephosphorylated by a unique mechanism compared to other MPKs. Consistent with this, there is no obvious KIM motif in RLPH2 (Labandera et al. 2018).

### 2.3 RLPH2 Preferentially Interacts With D‐Group MPKs

**FIGURE 2:**
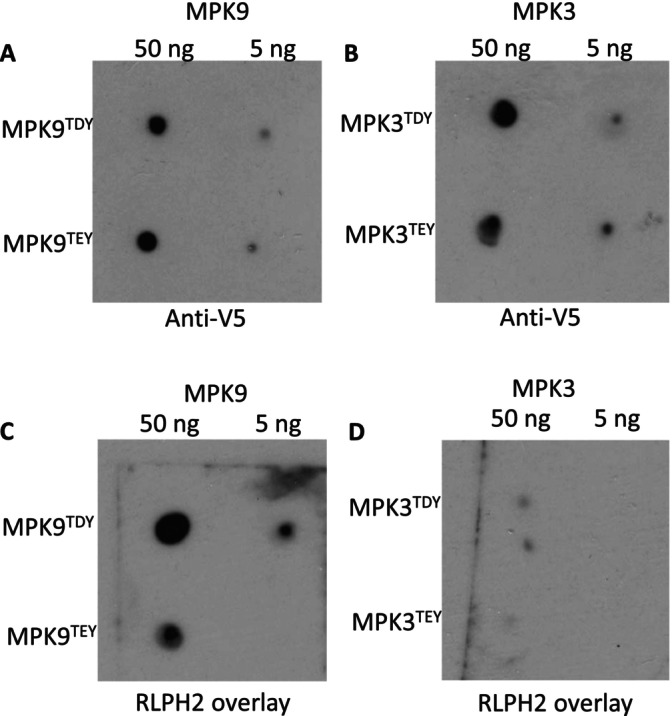
RLPH2 preferentially interacts with a D‐group MPK (MPK9). Purified MPK9 and MPK3 proteins, along with activation loop mutated versions MPK9^TEY^ and MPK3^TDY^ were spotted to a nitrocellulose membrane for overlay analysis with purified RLPH2 (1.5 μg/mL). Spotted MPK9 and MPK3 were probed with anti‐V5 for equal loading confirmation (A,B) or overlaid with RLPH2. Phosphatase binding was then detected with anti‐RLPH2 antibody (C,D). All experimentation was performed in parallel to ensure comparability and in triplicate.

With phospho‐proteomics having identified the D‐group MPKs as putative substrates of RLPH2, we chose MPK9 as a representative of the D‐group MPKs and compared it to the A‐group enzyme, MPK3. We began by performing overlay dot blots to explore the affinity of the bacterially expressed RLPH2‐V5‐His6 for the full‐length, purified, and activated/phosphorylated MPK9‐V5‐His6 and MPK3‐V5‐His6 and the potential role of the X amino acid in their TXY motif (Figure 2). For this, the MPKs were also mutated at the TXY with their counterpart amino acid (TDY to TEY and TEY to TDY). RLPH2 readily binds the TDY‐containing MPK9 (MPK9^TDY^) but shows reduced association if the activation loop TDY aspartate is mutated to a glutamate (MPK9^TEY^) (Figure 2C). RLPH2 displayed almost no binding to the TEY‐containing A‐group MPK, MPK3 (MPK3^TEY^), and changing the TEY motif glutamate to aspartate (MPK3^TDY^) did not increase affinity (Figure 2D). This suggests that the TDY motif plays a role in binding/substrate specificity; however, that single amino acid change is not enough, and another feature of the D‐group enzymes provides docking specificity. Notably, only the D‐group enzymes have an extended C‐terminal tail.

### 2.4 RLPH2 Preferentially Dephosphorylates D‐Group MPKs

**FIGURE 3:**
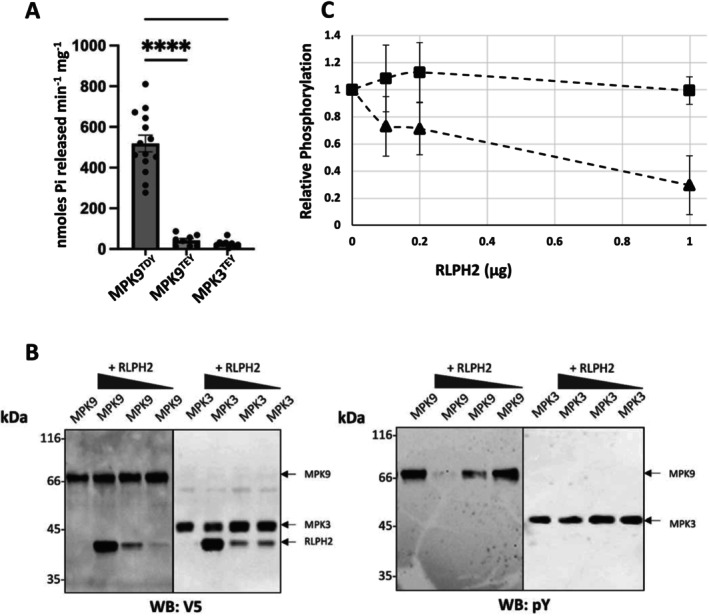
RLPH2 preferentially dephosphorylates D‐group (MPK9) MPKs. (A) Purified RLPH2 was used for in vitro phosphopeptide assays based on the dually phosphorylated activation loops of *Arabidopsis* MPK9^TDY^ (GLARVSFNDAPSAIFWpTDpYVATR), mutated MPK9 (MPK9^TEY^) (GLARVSFNDAPSAIFWpTEpYVATR), and MPK3^TEY^ (DFGLARPTSENDFMpTEpYVVTR) where pT and pY are phospho‐threonine and phospho‐tyrosine, respectively. Shown is the mean where each assay was done in duplicate and repeated either 15 times for MPK9^TDY^ or 8 times for MPK9^TEY^ and MPK3^TEY^. Comparison of MPK9^TDY^ to MPK9^TEY^, and MPK3^TEY^ was performed using a statistical one‐way ANOVA, showing a significant difference between MPK9^TDY^ and all other assays (*p* < 0.0001; one‐way ANOVA). (B) Varying amounts of RLPH2 was used to dephosphorylate recombinant MPK9^TDY^ (MPK9) or MPK3^TEY^ (MPK3) in vitro that was dually phosphorylated prior to the assay. One blot (left) was used to show equal loading using the V5 tag (WB: V5), whereas the other (right) monitored phosphorylation status of the pY after the assay (WB: pY). Representative blots are shown in (B), whereas in (C) the relative phosphorylation from three sets of blots is shown (MPK9, triangles; MPK3, squares). Here, the signal intensity of the pY signal is plotted relative to the total protein (V5) signal. Mass standards are shown in kDa.

Next, we examined the ability of RLPH2 to dephosphorylate phosphopeptides derived from the activation loop of MPK9 (Group D MPK) and MPK3 (Group A MPK). Previously, we demonstrated that RLPH2 has an enhanced V_max_ and decreased K_m_ for a human MPK ERK1/2 derived activation loop dually phosphorylated peptide substrate (pTxpY), compared to the same peptide solely phosphorylated on tyrosine (pY) (Labandera et al. 2018). Using dually phosphorylated peptides derived from the activation loops of MPK9 and MPK3, RLPH2 displayed 19‐fold higher activity for the MPK9 substrate versus MPK3 (Figure 3A). One of the key differences between the MPK9 (GLARVSFNDAPSAIFWpTDpYVATR) and MPK3 (DFGLARPTSENDFMpTEpYVVTR) activation loop peptides is the D versus E in the TXY motif (see Figure S2). When we altered the MPK9 peptide solely at the TXY motif by converting the aspartate (D) to glutamate (E) (GLARVSFNDAPSAIFWpTEpYVATR; MPK9^TEY^), we observed a remarkable ~12‐fold decrease in activity (Figure 3A). This indicates that much of RLPH2's substrate specificity comes from this single amino acid change and supports the idea that RLPH2 specifically targets D‐group MPKs in vivo, as suggested from the phospho‐proteomic dataset (Table 1).

Next, we examined the ability of RLPH2 to dephosphorylate full‐length recombinant tagged MPK9 or MPK3 in vitro. MPK3 proteins were phosphorylated by the upstream kinase (CKK4DD [Lampard et al. 2014]) and MPK9 proteins were allowed to autophosphorylate, resulting in active, dual phosphorylated (pTxpY) (Nagy et al. 2015) MPKs (Figure S4). Initially, we performed an in vitro dephosphorylation assay of each MPK with 5 μg of RLPH2, and as shown in Figure S4a–c, RLPH2 completely dephosphorylated the MPK9 pY, but not the MPK3 pY or the pT of either enzyme. Next, we explored the same dephosphorylation at varying and lower amounts of RLPH2. As shown in Figure 3B,C, RLPH2 again readily dephosphorylated the pY of MPK9 but did not dephosphorylate the pY of MPK3. Additional assays using WT MPK3 (MPK3^TEY^) and MPK9 (MPK9^TDY^) or with switched TDY and TEY motifs demonstrate that altering D for E in MPK9 (MPK9^TEY^) drastically reduces the ability of RLPH2 to dephosphorylate MPK9 (Figure S4d), whereas the reverse change on MPK3 (E to D; MPK3^TDY^) did not increase dephosphorylation of MPK3. Again, RLPH2 had little to no ability to dephosphorylate the pT of any TXY motif (Figure S4c,e) (Labandera et al. 2018), further supporting the notion that RLPH2 is a D‐group MPK tyrosine specific phosphatase.

### 2.5 D‐Group MPKs Bind PP1

Our data indicates that RLPH2 specifically binds D‐group MPKs and selectively dephosphorylates the activation loop pY of these enzymes. This is facilitated by recruiting the MPK via docking the pT in the RLPH2 basic pocket adjacent to the active site (Labandera et al. 2018). However, complete inactivation of MPKs typically requires the dephosphorylation of both TXY motif residues, which RLPH2 does not appear to do. Interestingly, as discussed above, the D‐group MPKs possess an insert in their activation loop (Figures S2 and S3) containing a potential docking site for a serine/threonine specific phosphatase, PP1 ( *A. thaliana* PP1 isoforms are referred to as TOPP1‐TOPP9 [Uhrig et al. 2013]). In eukaryotes, the RVXF motif functions as a PP1 recruitment motif, allowing the phosphatase to be regulated by a series of protein interactors that direct its function in the cell by guiding PP1 catalytic subunits to their corresponding substrates (Brautigan and Shenolikar 2018; Uhrig et al. 2013; Bollen et al. 2010). A subset of PP1 interactors also have additional binding residues C‐terminal of the RVXF motif (RVxF_5‐8_ΦΦ_8‐9_R, where Φ is a hydrophobic residue) (Choy et al. 2014), a feature also present in D‐group MPKs (Figure S3). This, combined with the fact that activation loops are on the surface of protein kinases, supports the hypothesis that these D‐group MPKs also interact with PP1 through the RVXF motif to potentially drive dephosphorylation of the pT residue of the TDY motif, or to target other phosphorylation sites in other proteins, or even the abundant phospho‐sites of the C‐termini of D‐group MPKs (Nagy et al. 2015; Mergner et al. 2020). Importantly, the RVXF motif insert found in D‐group MPK activation loops is not found in the Group A, B, or C MPKs (Figure S2). Further analyses of plant and other eukaryotic protein kinases found no evidence for any other protein kinase activation loops, in any organism, to have this PP1 docking insert, making this another unique attribute of plant D‐group MPKs. Given the conservation of the novel TDY activation loop and the putative PP1 binding site across plant D‐group MPKs [see additional file 4 in Ichimura et al. (2002) and our observations], this evidence suggests that docking PP1 is fundamental to the function of D‐group enzymes.

**FIGURE 4:**
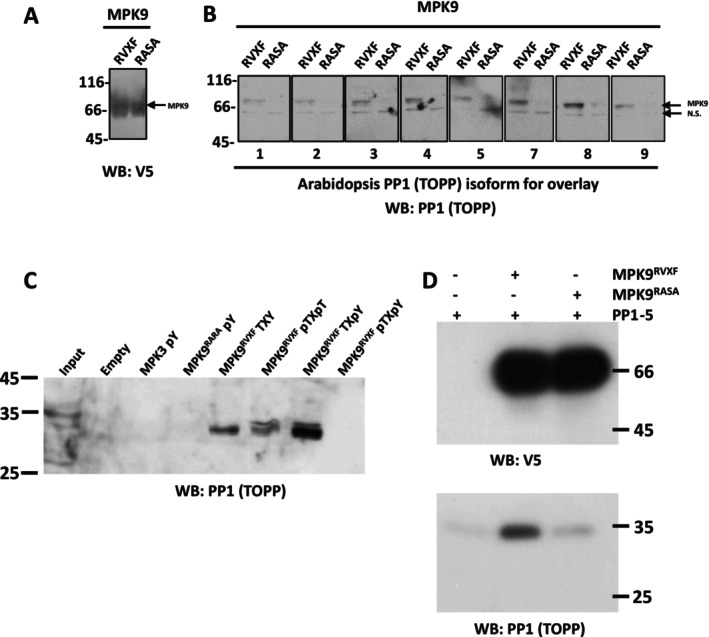
PP1 (TOPP) binds the D‐group MPK9 activation loop. (A) Purified MPK9 or MPK9 with mutated putative PP1 binding RVSF motif (MPK9^RASA^) was blotted with anti‐V5 tag antibody to show equal loading. Equivalent amounts of each sample was loaded in the lanes labeled RVSF and RASA in Panel B. (B) Purified MPK9^RVSF^ or MPK9^RASA^ was incubated (overlaid) with purified recombinant *Arabidopsis* PP1 isoforms (TOPPs) 1 through 9 (except PP1 isoform 6) as indicated below each box. Binding of PP1 to MPK9 was visualized with an anti‐PP1(TOPP) antibody. N.S. is a nonspecific band detected by the PP1 antibody in all samples (*n* = 3). (C) An *Arabidopsis thaliana* clarified extract (input) was incubated with MPK3 pY peptide (DFGLARPTSENDFMTEpYVVTR), MPK9 peptide (GLARVSFNDAPSAIFWpTDpYVATR) and MPK9 variants as indicated, where pT and pY are phospho‐threonine and phospho‐tyrosine, respectively. Empty is beads alone. SDS‐eluted beads were blotted for PP1 binding. (D) Pulldown assays were performed with MPK9^RVxF^ and MPK9^RASA^ (5 μg) with *Arabidopsis* PP1 isoform 5 (1 μg, TOPP5) as the representative PP1 isoform. MPK9 was detected with a V5 epitope antibody and PP1 with a pan‐TOPP1 isoform antibody (Templeton et al. 2011). Experimentation with *Arabidopsis* PP1 isoform 6 (TOPP6) was not performed as it does not bind and purify on microcystin‐Sepharose. Mass standards are shown in kDa, *n* = 3.

Given these observations, we proceeded to explore whether PP1 indeed binds to D‐group MPKs. To do this, we first investigated an MPK9 model generated by AlphaFold2. As predicted, the RVXF motif is surface exposed (Figure S5). We then expressed and purified each TOPP (TOPP1–TOPP9 [Templeton et al. 2011]) using the affinity matrix microcystin‐Sepharose (Lyons et al. 2018) and performed overlays on each TOPP using purified MPK9 where the putative PP1 binding RVXF motif was maintained or mutated to RASA (non‐PP1 binding mutations [Uhrig et al. 2013; Bollen et al. 2010]). This demonstrated that each PP1 isoform can bind MPK9, and this is dependent on the key hydrophobic residues of the RVXF sequence (Figure 4A; note that *Arabidopsis* PP1 isoform 6 does not bind to microcystin and thus did not purify on the affinity matrix [Templeton et al. 2011]). We then used (P)‐peptides derived from MPK9 and MPK3 activation loops to test their ability to bind PP1 isoforms from an *Arabidopsis* extract. As shown in Figure 4B, PP1 fails to be recruited by the MPK3 peptide(s) but is enriched on the MPK9 (P)‐peptides, in particular the TxpY peptide. Interestingly, when the MPK9 peptide is dual phosphorylated (pTXpY), PP1 is not retained, for reasons we have yet to understand. Next, we used *Arabidopsis* PP1 isoform 5 as a representative to perform an in vitro pulldown between purified MPK9 and AtPP1. As predicted, this association was dependent upon the RVXF motif (Figure 4C).

### 2.6 RLPH2 Represses Seed Dormancy Release and Affects Seed Sensitivity to GA Synthesis Inhibitors and ABA

**FIGURE 5:**
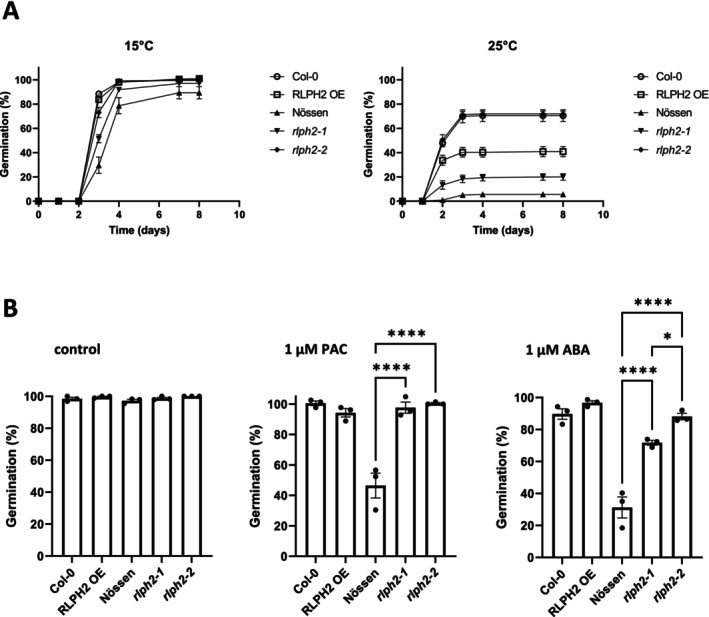
Germination phenotypes of RLPH2 knockout and overexpressing seed lines. (A) Seeds from wild‐type Nössen (solid triangle), *rlph2‐1* (solid triangle), *rlph2‐2* (solid diamond), Col‐0 (open circle) and *35S::*RLPH2‐cTAP (*RLPH2‐OE*) (open square) were incubated at 15°C and 25°C in darkness for 8 days. Results represent the mean germination percentage ± SE of four replicates of 50 seeds. (B) Assessment of ABA and paclobutrazol (PAC) sensitivity of non‐dormant seeds. Here, Nössen, *rlph2‐1*, *rlph2‐2*, Col‐0, and *RLPH2‐OE* seeds were stratified 4 days at 4°C on MS agar medium (control) or in the presence of 1 μM ABA or 1 μM PAC. The germination rate was scored after 7 days on light at 21°C. Results represent the mean germination percentage ± SE of three replicates of 50 seeds. Asterisks indicate statistical differences between Nössen and *rlph2* mutants or between Col‐0 and *RLPH2‐OE*, as determined by one‐way ANOVA with post hoc Tukey's test (*p* < 0.05).

Few D‐group MPKs have known defined nonredundant functions, with one exception being AtMPK8, which has been characterized in promoting seed germination (Zhang et al. 2019). Therefore, to address a possible role of RLPH2 in seed germination, freshly harvested RLPH2‐OverExpressor (*RLPH2‐OE*) (Columbia ecotype) and *rlph2* (Nössen ecotype) knockout seeds were germinated together with their respective WT lines at 15°C (Figure 5A). In these conditions, the germination of all seed lines was over 89% after 7 days. The *rlph2‐1* and *rlph2‐2* mutant lines exhibited accelerated germination compared to the WT Nössen background. To compare the dormancy level of the different seed lines at harvest, germination was also performed at 25°C (Figure 5A). In these conditions, *rlph2* mutant seeds presented significantly higher germination than WT Nössen seeds (5.6%), with *rlph2‐2* germinating better than *rplh2‐1* (72% and 20%, respectively). Pairwise *t*‐test of *rlph2‐1* and *rlph2‐2* versus WT Nössen at 8 days were significant (*p* < 0.0034 and *p* < 0.0001, respectively). Conversely, the RLPH2 overexpressing line (*RLPH2‐OE*) presented a lower germination rate compared with WT Col‐0 seeds (41% and 70.4%, respectively). These data indicate that RLPH2 is a positive regulator of seed dormancy.

Next, the sensitivity of the different seed lines to ABA and to the GA synthesis inhibitor paclobutrazol (PAC) was compared after 7 days of germination. Dormancy was released by stratification and all seeds fully germinated in the absence of treatment (Figure 5B). In contrast, in the presence of 1 μM ABA, *rlph2‐1* and *rlph2‐2* seeds exhibited a higher germination rate than WT Nössen seeds (71.7%–88.1% and 31.2%, respectively). At higher ABA concentrations, the germination of the three lines was almost fully inhibited (Figure S6). On the other hand, *rlph2* mutants were less affected by 1 μM PAC compared to WT Nössen seeds (97.6%–100% and 46.4% germination, respectively) (Figure 5B). This difference was also observed at higher PAC concentrations (Figure S7). No difference in sensitivity to ABA and PAC was observed between WT Col‐0 and *RLPH2‐OE* seeds at any concentration (Figures 5B, S6, and S7). These data suggest that the function of RLPH2 during seed germination involves ABA and GA pathways.

## 3 Discussion

MPKs are ubiquitous eukaryotic signaling proteins. In all cases, they are activated by phosphorylation of their activation or T‐loops within a characteristic TXY motif (Peti and Page 2013). This event, as in all protein kinases, reshapes the active site, repositioning key catalytic residues (Taylor and Kornev 2011). In plants, the MPK family has expanded compared to yeast and humans (Ichimura et al. 2002; Bardwell et al. 2009) and in *Arabidopsis* includes 20 members (Figure S1). These members form four groups based on sequence, with the D‐group having unique sequence additions.

In this study, we provide evidence that the plant tyrosine phosphatase RLPH2 specifically targets and dephosphorylates the pY residue of the TDY motif in D‐group MPKs. These enzymes are unique in possessing a TDY activation loop motif, a PP1 docking SLiM in the same loop, and a C‐terminal extension with multiple mapped phosphorylation sites (Nagy et al. 2015). The specificity of RLPH2 toward the D‐group MPKs seems to involve two components: (1) In an overlay assay, RLPH2 readily binds MPK9^TDY^, and this is marginally reduced when converted to MPK9^TEY^. (2) RLPH2 does not bind the A‐group MPK3 regardless of the T‐loop motif being TEY or TDY, suggesting that this interaction is driven by some feature outside of the TXY motif—perhaps the C‐terminal tails of the D‐group MPKs. Indeed, alignment of the D‐group MPK C‐terminal tails reveals a conserved core sequence (Figure S8). In dephosphorylation assays using phosphopeptides, changing the D to an E in the MPK9 substrate peptide drastically reduces activity, whereas the MPK3^TEY^ phospho‐peptide is simply a poor RLPH2 substrate in comparison, suggesting substrate sequence specificity is conferred by the active site. This is supported by dephosphorylation assays of the recombinant enzymes leading to a model where RLPH2 achieves D‐group specificity by associating with some structural feature unique to D‐group MPKs; then a second layer of specificity is driven by the active site itself, selecting for TDY motifs.

Classically, it has been believed that full activation and inactivation of MAPKs require dual TXY phosphorylation and dephosphorylation, respectively; however, the mono‐phosphorylated versions have been detected in human cells (Zhang et al. 1993) and may serve as reservoirs of MAPK that can be rapidly converted to fully activated in cells (Zhang et al. 1993). Most MAPKs are dephosphorylated by specific MAPK phosphatases that target both the T and Y of the loop, yet human dual specificity phosphatase VHR/DUSP3, like RLPH2, recruits specific MAPKs via a pT binding basic pocket to specifically dephosphorylate solely the pY (Schumacher et al. 2002). The mono‐phosphorylated product could then potentially be acted upon by specific PP2C (Zhang et al. 2008) or PP2A (Zhang et al. 2008; Peti and Page 2013; Alessi et al. 1995) serine/threonine phosphatases. The activity of the mono‐phosphorylated MAPKs seems to vary depending on the enzyme, based on the few detailed studies. For instance, the protein kinase activity of human p38/pY and p38/pT is about 1000‐ and 10‐fold lower than that of the dual phosphorylated p38/pTXpY, respectively, whereas mono‐phosphorylated (pY or pT) ERK2 displays activities intermediate of full and non‐phosphorylated enzyme (Zhang et al. 2008). This led to the speculation that mono‐phosphorylated versions could have unique substrate specificity/biological roles or, as stated, act as a reservoir to rapidly activate to fully phosphorylated enzyme (Zhang et al. 2008). Whether plant D‐group MPKs only phosphorylated on threonine (pT) display these properties is unknown and is worthy of further future investigation.

Observationally, we identified a putative PP1 binding SLiM in all D‐group MPK activation loops (a feature absent in A‐, B‐, and C‐group MPKs), and consistent with being surface exposed, we show this sequence does bind serine/threonine phosphatase 1 (PP1). Interestingly, through the use of the PPP phosphatase inhibitor okadaic acid, the activation loop of MPK9 was shown to be regulated in vivo by an okadaic acid sensitive phosphatase (PP2A, PP1, and PP4–PP6) (Nagy et al. 2015), and we previously showed that RLPH2 activity is not affected by this inhibitor in vitro (Uhrig et al. 2016). We therefore speculate that the recruited PP1 may dephosphorylate the pT of the MPK T‐loop itself, may target the C‐terminal tail phosphorylation sites (Nagy et al. 2015; Mergner et al. 2020), or could simply target other proteins linked to D‐group MPK function. Interestingly, as shown through peptide pulldowns, PP1 does not bind the T‐loop of MPK9^TDY^ if the TDY motif is dually phosphorylated (pTDpY). This could suggest a temporality in the mechanism of dephosphorylation of the D‐group MPKs. It is also worth noting that five of the eight D‐group activation loop RVXF motifs have a serine in the X‐position (RVSF; Figure S2) and phosphorylation of this site would block PP1 binding (Nasa et al. 2018), as has been shown for RVSF motifs in other PP1 interactors, potentially bringing another level of regulation to D‐group enzyme regulatory events.

Finally, we began exploring RLPH2 function *in planta*. Looking for the already known functions of the D‐group MPKs, we noticed their functions have not been well studied, with one exception being AtMPK8, which has previously been linked to the promotion of seed dormancy release. If RLPH2 regulates D‐group MPKs, we reasoned that loss of RLPH2 could perhaps keep D‐group MPKs phosphorylated and therefore active, promoting germination. The germination capacity of *rlph2‐1* and *rlph2‐2* revealed accelerated germination rates; therefore, RLPH2 acts as a repressor of germination, perhaps through keeping select D‐group MPKs dephosphorylated on T‐loop tyrosine. Due to GA involvement in dormancy release, we investigated the involvement of RLPH2 in GA metabolism. Both *rlph2* mutants were significantly less sensitive to the GA biosynthesis inhibitor PAC, suggesting that RLPH2 is a negative regulator of seed germination by either negatively regulating GA synthesis or signaling or positively regulating GA catabolism. With seed germination orchestrated by a balance of GA–ABA signaling, ABA sensitivity of *rlph2* mutants was also assessed. Both lines were significantly less sensitive to 1 μM ABA than the WT Nössen plants. This suggests that RLPH2 is also involved in negatively regulating seed germination by being a positive regulator of ABA biosynthesis/signaling or a negative regulator of ABA catabolism. If RLPH2's function in the regulation of germination is related to MPK8, it is yet to be investigated.

In conclusion, we have revealed the D‐group MPKs as targets of the novel plant tyrosine phosphatase RLPH2 and uncovered that its substrate specificity relies in part on a single amino acid substitution (D or E in the TXY motif) in substrates. We also demonstrated the unique ability of D‐group MPKs to recruit PP1 via a conserved RVXF SLiM, and it is dependent on the phosphorylation status of the TDY motif. Finally, we find that RLPH2 is involved in the inhibition of seed germination in a GA‐ and ABA‐dependent manner.

## 4 Materials and Methods

### 4.1 Phospho‐Proteomics Sample Preparation

T‐DNA insertion mutant lines for *rlph2‐1* (RATM‐13‐2130‐1_G) and *rlph2‐2* (RATM‐ 13‐3204‐1_G) were obtained from RIKEN and are in a Nössen background. WT *A. thaliana* Nössen and two independent *rlph2* (*rlph2‐1* and *rlph2‐2*) mutant allele containing *A. thaliana* plant lines were grown as described in Uhrig et al. (2016). Rosette leaves were harvested, flash‐frozen in liquid nitrogen, and stored at −80°C until day of use. Protein extraction and digestion was performed by Filter Aided Sample Preparation (FASP) as previously described without deviation (Uhrig et al. 2019). Phosphorylated peptides were then enriched using TiO_2_ beads, at a concentration of 10 mg/mL as previously described (Uhrig et al. 2021). Eluate phosphopeptides were then acidified with formic acid to a concentration of 0.5% (v/v) and dried with speed vacuum (Thermo Fisher). Dried phosphopeptides were then resuspended in 800 μL ice‐cold IP buffer (50 mM HEPES‐NaOH pH 7.4, 50 mM NaCl), pH adjusted to 7.5 and incubated end‐over‐end, overnight at 4°C with 50 μL of anti‐phospho‐tyrosine antibody agarose beads (Santa Cruz, sc‐7020) and 15 μL of anti‐phospho‐tyrosine antibody Sepharose beads (p‐Tyr‐100, Cell Signaling, #7902) pre‐equilibrated in IP buffer (*n* = 2 independent enrichments from separate batches of plants). Beads were pelleted and washed three times for 2 min at RT with 1 mL IP buffer, followed by three washes with ddH_2_O. Enriched tyrosine phosphorylated peptides were then eluted with 3 × 200 μL of an 80% (v/v) ACN/2% (v/v) formic acid (FA) solution. Eluates were pooled and dried by speed vacuum (Thermo Fisher).

### 4.2 Mass Spectrometry Analysis

All dried phosphopeptide samples were dissolved in a 3% ACN (v/v) / 0.1% (v/v) TFA solution and desalted using ZipTip C18 pipette tips (Millipore; ZTC18S960) as previously described (Uhrig et al. 2019). Phosphopeptides were then dried and dissolved in a solution of 3.0% ACN (v/v) / 0.1% FA (v/v) prior to mass spectrometry analysis. Dissolved samples were then injected using an Easy‐nLC 1000 system (Thermo Fisher) and separated on a 50 cm EasySpray nano‐LC column (ES803). The column was equilibrated with 100% solvent A (0.1% FA in water). Peptides were eluted using the following gradient of Solvent B (0.1% FA in ACN): 0–50 min; 0%–25% B, 50–60 min; 25%–32% B, 60–70 min; 32%–98% B at a flow rate of 0.3 μL/min. High accuracy mass spectra were acquired using an Orbitrap Fusion mass spectrometer (Thermo Fisher) that was operated in data‐dependent acquisition mode. All precursor signals were recorded in the Orbitrap using quadrupole transmission in the mass range of 300–1500 m/z. Spectra were recorded with a resolution of 120,000 at 190 m/z, a target value of 4E5, and the maximum cycle time was set to 3 s. Data dependent MS/MS were recorded in the linear ion trap using quadrupole isolation with a window of 2 Da and HCD fragmentation with 30% fragmentation energy. The ion trap was operated in rapid scan mode with a target value of 3E4 and a maximum injection time of 100 ms. Precursor signals were selected for fragmentation with a charge state from +2 to +7 and a signal intensity of at least 1E4. A dynamic exclusion list was used for 30 s, and maximum parallelizing ion injections was activated. After data collection, peak lists were extracted from the instrument raw files using Proteome Discoverer (Version 1.4/2.1) and exported to mgf format for subsequent database analysis. All MS/MS samples were analyzed using Mascot (Matrix Science, Version 2.5.3).

Tandem MS spectra were then searched with Mascot 2.5 (http://www.matrixscience.com) Matrix Science, London, UK) against The Arabidopsis Information Resource (TAIR10) protein database using a concatenated decoy database supplemented with contaminants. Mascot search parameters included trypsin specificity, peptide mass tolerance of 10 ppm, MS/MS tolerance of 0.6 Da, charge state of +2, +3, or +4, and fixed cysteine carbamidomethylation. Variable modifications included serine, threonine, and tyrosine phosphorylation and methionine oxidation with one missed cleavage. Mascot identifications were then imported into Scaffold Q + S (http://www.proteomesoftware.com) and filtered for a protein and peptide false discovery rate (FDR) ≤ 0.01 (Table S2). Scaffold data were then assessed for pY phosphoprotein identifications unique to *rlph2‐1* and *rlph2‐2* (Table S2).

### 4.3 Cloning, Expression and Purification of Enzymes

RLPH2 was expressed and purified as in Uhrig et al. (2016). Synthesized Arabidopsis MPK9 (At3g18040) was codon optimized for bacterial expression (GenScript). MPK9 was then cloned into bacterial expression vector pDEST42 (Invitrogen) for expression using BL21 (DE3) CodonPlus‐RIL *Escherichia coli* . The resulting MPK9‐V5‐His6 construct was then produced in BL21 (DE3) CodonPlus‐RIL *E. coli* as follows: Bacteria were grown at 37°C until an OD_600_ of 0.4–0.6, followed by induction with 0.1 mM IPTG for 7 h at 37°C. Induced cells were centrifuged at 3300 × g for 20 min, with the resulting cellular pellet resuspended in 15 mL of resuspension buffer (50 mM HEPES pH 7.5, 150 mM NaCl, 5% v/v glycerol, 10 mM imidazole) plus 1 mM benzamidine and 1 mM PMSF. Bacterial cells were then lysed by French press three times at 1000 psi, followed by centrifugation at 50,000 × g for 45 min. The resulting supernatant was then collected and filtered through miracloth before a 1‐h incubation with 1 mL of Ni‐NTA agarose (Qiagen). Matrix was washed with Buffer A (50 mM HEPES pH 7.5, 1 M NaCl, 5% glycerol, 10 mM imidazole, 0.5% Tween 20), followed by Buffer B (50 mM HEPES pH 7.5, 150 mM NaCl, 5% glycerol, 10 mM imidazole) before being eluted with 400 mM imidazole in 50 mM HEPES pH 7.5, 150 mM NaCl, 5% glycerol. Purified protein was then concentrated in an Amicon 30 kDa centricon (Millipore, UFC9030), and buffer was exchanged for wash buffer B. Protein concentration was determined by Bradford using BSA as standard and enzyme was stored at −80°C until use.

*A. thaliana* MPK3 (At3g45640) in pDEST42 (MPK3‐V5‐His6) was purified, concentrated, and stored like MPK9, except bacteria were only induced for 4 h. A constitutively active CAMKK4‐GST for MPK3 phosphorylation and activation was expressed in a BL21 (DE3) CodonPlus‐RIL *E. coli* strain (Lampard et al. 2014). Cells were grown until an OD_600_ of 0.4–0.6 was reached, at which point 0.1 mM IPTG was added to induce expression for 6 h at 30°C. Cells were centrifuged at 3300 g for 20 min at 4°C, after which the pellet was resuspended in 15 mL of resuspension buffer (50 mM HEPES pH 7.5, 150 mM NaCl, 3 mM DTT, 0.05% NP‐40, 2 μg/mL leupeptin, 5 μg/mL pepstatin) and lysed by French press, followed by centrifugation at 50,000 g for 45 min. The supernatant was collected and filtered through miracloth before a 1‐h incubation with 1 mL of glutathione‐Sepharose 4B matrix (Cytiva). Matrix was washed with 300 CV of wash buffer C (50 mM HEPES pH 7.5, 750 mM NaCl, 1 mM DTT, 0.1% NP‐40), followed by an additional 100 mL of wash buffer D (50 mM HEPES pH 7.5, 150 mM NaCl, 1 mM DTT) before elution with 20 mM reduced glutathione in 50 mM HEPES pH 7.5, 150 mM NaCl, and 1 mM DTT. Protein was concentrated as for MPK9.

The *Arabidopsis* PP1 enzymes (TOPP1–TOPP9) were purified on microcystin‐Sepharose as described (Templeton et al. 2011). TOPP6 was not purified because it is not microcystin sensitive.

### 4.4 Site‐Directed Mutagenesis

Using the QuikChange Lightning Site‐Directed Mutagenesis Kit (Agilent Technologies), mutants of MPK9 and MPK3 were generated by point mutations in their respective activation loops. The MPK9^RASA^ mutant construct had valine and phenylalanine of the RVSF motif (see Figure S2) changed to alanine. The MPK9^TEY^ mutant construct had the aspartic acid (D) within MPK9's TDY motif switched for a glutamic acid (E). Similarly, the MPK3^TDY^ mutant construct had its glutamic acid in the TEY motif switched for an aspartic acid. Primers are shown in Table S3.

### 4.5 Phosphorylation of MPK9 and MPK3 Activation Loops

Purified MPK9, MPK3, and activation loop mutant enzymes were diluted to 500 μL in 25 mM HEPES pH 7.5, 10 mM NaCl, 15 mM MgCl_2_, and 5 mM ATP. MPK9 was allowed to autophosphorylate, or 500 ng of constitutively active protein kinase CAMKK4(DD) was added to MPK3, and samples were incubated at 30°C. After 2 h, Mg‐ATP was removed by concentration to 50 μL and dilution back to 500 μL with 50 mM HEPES pH 7.5, 150 mM NaCl, 5% glycerol using a 30 kDa centricon, five times. Protein samples were then stored at −80°C until the day of use.

### 4.6 RLPH2 Phosphatase Assays

For phosphopeptide assays, purified RLPH2 (1 μg) was incubated for 30 min at 200 rpm at 30°C in 160 μL of assay buffer (50 mM HEPES pH 7.5, 150 mM NaCl) containing 0.5 mM peptide substrate in a 96‐well plate. Peptides were at least 95% pure and purchased from GL Biochem (Shanghai) Ltd. Control assays were performed in parallel with identical conditions but lacking enzyme. The reaction was stopped with the addition of 40 μL malachite green solution, and OD was measured at 630 nm using a plate reader. The activity of RLPH2 was measured by comparing absorbance at 630 nm with the standard curve of free Pi prepared in parallel.

The dephosphorylation of purified recombinant and phosphorylated MPK9 and MPK3 (5 μg) was incubated in the absence of or with 5, 1, 0.2, and 0.1 μg RLPH2 in 20 mM HEPES pH 7.5, 10 mM NaCl, 0.1 mM DTT for 1 h 30 min at 30°C. Reactions were stopped by the addition of 5X SDS cocktail and heated at 95°C for 10 min. Dephosphorylation was monitored by blotting with phospho‐specific antibodies.

### 4.7 Western and Far‐Western Blots

For MPK proteins used in RLPH2 dephosphorylation assays, samples were run on a 10% SDS‐PAGE and then visualized by immunoblotting with either horseradish peroxidase (HRP) conjugated rabbit anti‐V5 (Immunology Consultants Laboratory, RV5‐45P‐Z) or anti‐pT (Cell Signaling Technologies, 42H4) and anti‐pY (Cell Signaling Technologies, PTyr‐100) for phospho‐status of the MPK protein. V5 antibody was diluted in 5% BSA and 0.1% TBST and incubated for 2 h and after washing imaged via the conjugated secondary antibody diluted 5000‐fold. The anti‐pT and anti‐pY primary antibodies (1:1000 and 1:5000, respectively) were diluted in 5% BSA and 0.1% TBST and incubated for 2 h and after washing probed with anti‐mouse secondary antibody diluted 5000‐fold. For PP1 far‐westerns (overlays), MPK9^RVXF^ and RVSF mutated RASA MPK9^RASA^ were run on SDS‐PAGE, blotted to nitrocellulose and probed with PP1 (TOPP) isoforms (2 μg/mL) for 4 h in TBS plus 5% milk powder as in Templeton et al. (Templeton et al. 2011). Antibodies to the TOPP isoforms were generated, affinity‐purified, and used as in (Templeton et al. 2011) to visualize PP1 binding. For dot‐blot far‐western, purified activated MPKs were dotted onto a nitrocellulose membrane, blocked and probed with anti‐V5 (for equal loading), or overlaid with purified RLPH2 (1.5 μg/mL) and detected with anti‐RLPH2 antibody as in Uhrig et al. (2016).

### 4.8 Peptide Pulldown Assays

Peptides from MPK9 (1 mg) containing the RVSF motif and different phosphorylation combinations on the TDY motif were coupled to CNBr‐Sepharose beads in coupling buffer (100 mM NaHCO3 pH 8.0, 500 mM NaCl) for 4 h at RT. Protein extracted from 10‐day‐old dark‐grown *Arabidopsis* cell culture was prepared as in Labandera et al. (2018), and 14 mg of clarified protein extract (including 25 mM NaF and 1 mM Na_3_VO_4_) was incubated with each peptide coupled bead for 3 h at 4°C. Beads were washed three times for 5 min with 50 mM HEPES‐NaOH pH 7.5, 150 mM NaCl, 25 mM NaF and 1 mM Na_3_VO_4_, 5% (v/v) glycerol, 0.1% (v/v) Tween 20, followed by one additional wash without Tween 20. Proteins were eluted with 2× SDS sample buffer and boiled for 5 min. Eluates were analyzed by western blotting with 3 μg/mL affinity purified TOPP antibody in 1% (w/v) BSA and TBS for 16 h at 4°C (Mergner et al. 2020).

### 4.9 TOPP5–MPK9 Pulldown Assay

TOPP5 (1 μg) and MPK9^RVXF^ and RVSF mutated MPK9^RASA^ (5 μg) were incubated together for 1 h at RT while simultaneously Ni‐NTA was incubated with 5 mg/mL BSA to block nonspecific interactions. Afterwards, TOPP5 alone or TOPP5 with MPK9^RVXF^ and MPK9^RASA^ was incubated with 0.2 mL Ni‐NTA for 1 h at RT, washed with 50 mM HEPES pH 7.5, 300 mM NaCl buffer, and 0.1% Tween 20, followed by buffer minus Tween 20, then eluted in 2× SDS cocktail to be analyzed by western blot as described above.

### 4.10 Germination Assays

For seed production, plants from the different genetic backgrounds (Col‐0, Nössen, RLPH2‐OE, *rlph2‐1*, and *rlph2‐2*) were grown simultaneously in a growth chamber (21°C; 16‐h light/8‐h darkness photoperiod; 100μE.m‐2.s‐1 light intensity). Seeds were collected 21 days after pollination, air‐dried for 7 days, and subsequently conserved at −20°C.

Germination of freshly harvested seeds was performed in darkness at 15°C or 25°C, as previously described (Leymarie et al. 2012). For ABA or PAC treatments, seeds were sown on half‐strength MS agar medium, supplemented with different concentrations of ABA or PAC, and stratified for 4 days at 4°C. Seeds were then incubated at 21°C under constant light, and germination was scored daily for 7 days.

## Author Contributions

The study was conceived by A.‐M.L., R.G.U., and G.B.M. Manuscript writing was a contribution of all authors. Mass spectrometry and analysis were performed by R.G.U. All biochemical experiments were a combined effort of A.‐M.L., R.T., S.M., J.J.J., and B.K. Sequence alignments and AlphaFold structure prediction were done by R.T., S.M., and J.J.J. Germination assays were performed by J.P. and E.B.

## Funding

This work was supported by the Natural Sciences and Engineering Research Council of Canada (NSERC) grants to G.B.M. and R.G.U., Alberta Innovates‐Technology Futures, and Open Doctoral Scholarship–University of Calgary Silver Anniversary Fellowship to A.‐M.L. E.B. and J.P. were supported by CNRS and Sorbonne Université.

## Conflicts of Interest

The authors declare no conflicts of interest.

## Peer Review

The peer review history for this article is available in the Supporting Information for this article.

## Supporting information


**Data S1:** Peer review.

**Table S1:** Plant proteins with pTxpY motifs extracted from Plant PTM Viewer (www.psb.ugent.be/webtools/ptm‐viewer/) (Willems et al. 2024).

**Table S2:** All Tyr phosphorylated peptides identified by Mascot and Scaffold Q + S.

**Table S3:** Primers used for site directed mutagenesis.

**Figure S1:** Phylogenetic analysis of the 20 MPK proteins in *Arabidopsis thaliana* . Groups A, B, C, and D are labeled with the corresponding MPKs present in each group. Sequence Viewer 8.0 was used to align the sequences and generate the phylogenetic tree.
**Figure S2:** Alignment of *Arabidopsis thaliana* MPK activation loops. Protein kinase activation loops are defined by the sequences DFG and APE. In MPKs, activation is achieved by phosphorylation of a TXY motif (green). *Arabidopsis* MPKs form four phylogenetically distinct groups as indicated (A–D) with only the D‐group have an aspartate (D) between the T and Y of the TXY motif, whereas Groups A–C have a E in this position (Ichimura et al. 2002). The D‐group enzymes also possess an insert in the loop that conforms to a protein phosphatase one (PP1) binding RVXF SLiM (blue).
**Figure S3:** *Arabidopsis thaliana* D‐group MPKs have an extended RVXF motif. The activation loops of the D‐group MPKs as defined by DFG and APE. Highlighted in blue are the amino acids associated with the extended RVXF motif, which starts with RVXF, then five to eight amino acids C‐terminal two hydrophobic residues (Val, Ile or Phe) and then eight to nine amino acid C‐terminal an arginine (R). Each D‐group enzyme conforms to this sequence motif further supporting the idea that these protein kinases recruit PP1.
**Figure S4:** RLPH2 solely dephosphorylates pY of MPK9^TDY^, but not MPK9^TEY^, MPK3^TEY^, or MPK3^TDY^. MPKs were dually phosphorylated in their activation loops (see Methods) and treated with or without purified RLPH2. Western blotting was performed to assess the MPK phospho‐status with either anti‐pY or pT antibodies. (A,D) Anti‐V5 immunoblot demonstrating equal loading. (B,C,E,F) Anti‐phospho‐tyrosine and anti‐phospho‐threonine immunoblots.
**Figure S5:** Predicted *Arabidopsis thaliana* MPK9 structure. The AlphaFold structure for MPK9 corresponding to UniProt ID Q9LV37 was downloaded from the AlphaFold Protein Structure Database (https://alphafold.ebi.ac.uk/). PyMol was used to visualize the structure and residues after tyrosine 376 were hidden for image clarity. Marked in pink is the activation loop, defined by DFG and APE, showing how the putative PP1 docking RVXF SLIM (light blue) is surface exposed. The activation loop TDY motif threonine (T) and tyrosine (Y) are presented with sidechains shown (dark blue).
**Figure S6:** Percentage germination after 7 days in the presence of ABA. ABA sensitivity of Nössen, *rlph2‐1*, *rlph2‐2*, Col‐0, and 35S::RLPH2‐Ctap line (*RLPH2‐OE*). Seeds were stratified 4 days at 4°C on MS agar medium without ABA (A, control) or in the presence of (B) 5 and (C) 20 μm ABA. The germination rate was scored after 7 days on light at 21°C. Results represent germination means ± SE of three replicates of 50 seeds. Asterisks indicate statistical differences between Nössen and *rlph2* mutants or between Col‐0 and the *RLPH2‐OE* line, as determined by one‐way ANOVA with post hoc Tukey's test (*p* < 0.05).
**Figure S7:** Percentage germination in the presence of PAC after 7 days. PAC sensitivity of Nössen, *rlph2‐1*, *rlph2‐2*, Col‐0, and 35S::RLPH2‐Ctap line (*RLPH2‐OE*). Seeds were stratified 4 days at 4°C on MS agar medium without PAC (A, control) or in the presence of (B) 3 and (C) 10 μm PAC. The germination rate was scored after 7 days on light at 21°C. Results represent germination means ± SE of three replicates of 50 seeds. Asterisks indicate statistical differences between Nössen and *rlph2* mutants or between Col‐0 and *RLPH2‐OE* line, as determined by one‐way ANOVA with post hoc Tukey's test (*p* < 0.05).
**Figure S8:** Conserved region of C‐terminal tails of *Arabidopsis thaliana* D‐group MPKs. The D‐group MPKs were aligned and the conserved region among the C‐terminal tails extracted and presented. The most conserved residues are highlighted in red.

## Data Availability

All raw data files and mass spectrometry parameters have been uploaded to ProteomeXchange (http://www.proteomexchange.org/) via the PRoteomics IDEntification Database (PRIDE; https://www.ebi.ac.uk/pride/). Project Accession: PXD038689. reviewer_pxd038689@ebi.ac.uk; Password: yqxu8Z7L. All other data are contained within the manuscript.
