# Supplementary material for: Phospho‐Proteomics Identifies D‐Group MAP Kinases as Substrates of the Arabidopsis Tyrosine Phosphatase RLPH2
Source: Plant Direct. 2026 Jan 20;10(1):e70137. doi: 10.1002/pld3.70137 (PMC12817480; doi:10.1002/pld3.70137)
Supplement: Supplementary file 4 — Table S3: Primers used for site directed mutagenesis. [file PLD3-10-e70137-s002.pdf]

Table S3. Primers used for site directed mutagenesis.

Mutant name	Mutation	Primer	Sequence (5'-3')
$MPK9^{TEY}$	D295E	Forward	CTTCTGGACCGAGTACGTGGCTACC
		Reverse	GGTAGCCACGTACTCGGTCCAGAAG
$MPK3^{TDY}$	E197D	Forward	CTACTTCAGAGAATGATTTTATGACTGATTATGTTGTTACGAGATGGTATAGAGCAC
		Reverse	GTGCTCTATACCATCTCGTAACAACATAATCAGTCATAAAATCATTCTCTGAAGTAG
$MPK9^{RASA}$	V282A/F284A	Forward	CGGTCTGGCGCGTGCTAGCGCTAACGATGCTCCGAG
		Reverse	CTCGGAGCATCGTTAGCGCTAGCACGCGCCAGACCG
