# Supplementary material for: Phospho‐Proteomics Identifies D‐Group MAP Kinases as Substrates of the Arabidopsis Tyrosine Phosphatase RLPH2
Source: Plant Direct. 2026 Jan 20;10(1):e70137. doi: 10.1002/pld3.70137 (PMC12817480; doi:10.1002/pld3.70137)
Supplement: Supplementary file 5 — Figure S1: Phylogenetic analysis of the 20 MPK proteins in Arabidopsis thaliana . Groups A, B, C, and D are labeled with the corresponding MPKs present in each group. Sequence Viewer 8.0 was used to align the sequences and generate the phylogenetic tree. Figure S2: Alignment of Arabidopsis thaliana MPK activation loops. Protein kinase activation loops are defined by the sequences DFG and APE. In MPKs, activation is achieved by phosphorylation of a TXY motif (green). Arabidopsis MPKs form four phylogenetically distinct groups as indicated (A–D) with only the D‐group have an aspartate (D) between the T and Y of the TXY motif, whereas Groups A–C have a E in this position (Ichimura et al. 2002). The D‐group enzymes also possess an insert in the loop that conforms to a protein phosphatase one (PP1) binding RVXF SLiM (blue). Figure S3: Arabidopsis thaliana D‐group MPKs have an extended RVXF motif. The activation loops of the D‐group MPKs as defined by DFG and APE. Highlighted in blue are the amino acids associated with the extended RVXF motif, which starts with RVXF, then five to eight amino acids C‐terminal two hydrophobic residues (Val, Ile or Phe) and then eight to nine amino acid C‐terminal an arginine (R). Each D‐group enzyme conforms to this sequence motif further supporting the idea that these protein kinases recruit PP1. Figure S4: RLPH2 solely dephosphorylates pY of MPK9TDY, but not MPK9TEY, MPK3TEY, or MPK3TDY. MPKs were dually phosphorylated in their activation loops (see Methods) and treated with or without purified RLPH2. Western blotting was performed to assess the MPK phospho‐status with either anti‐pY or pT antibodies. (A,D) Anti‐V5 immunoblot demonstrating equal loading. (B,C,E,F) Anti‐phospho‐tyrosine and anti‐phospho‐threonine immunoblots. Figure S5: Predicted Arabidopsis thaliana MPK9 structure. The AlphaFold structure for MPK9 corresponding to UniProt ID Q9LV37 was downloaded from the AlphaFold Protein Structure Database (https://alphafold.ebi.ac.uk/) [file PLD3-10-e70137-s001.pdf]

Title of the article:

Phospho-proteomics identifies D-group MAP kinases as substrates of the Arabidopsis tyrosine phosphatase RLPH2

Authors:

Anne-Marie Labandera, Ryan Toth, Sierra Mitchell, Jayde J Johnson, Brooklyn Kurucz, Juliette Puyaubert, Emmanuel Baudouin, R. Glen Uhrig and Greg B Moorhead

List of the material included:

Fig. S1. Phylogenetic analysis of the 20 MPK proteins of Arabidopsis thaliana.

Fig. S2. Activation loops of Arabidopsis thaliana MPKs.

Fig. S3. D group MPKs have an extended RVXF motif.

Fig. S4. RLPH2 solely dephosphorylates pY of MPK9 TDY, but not MPK9 TEY, MPK3 TEY or MPK3 TDY.

Fig S5. Arabidopsis thaliana MPK9 structure

Fig. S6. Percentage germination after 7 days in the presence of ABA.

Fig. S7. Percentage germination in the presence of PAC after 7d.

Fig. S8. Conserved region of C-terminal tails of Arabidopsis D-group MPKs.

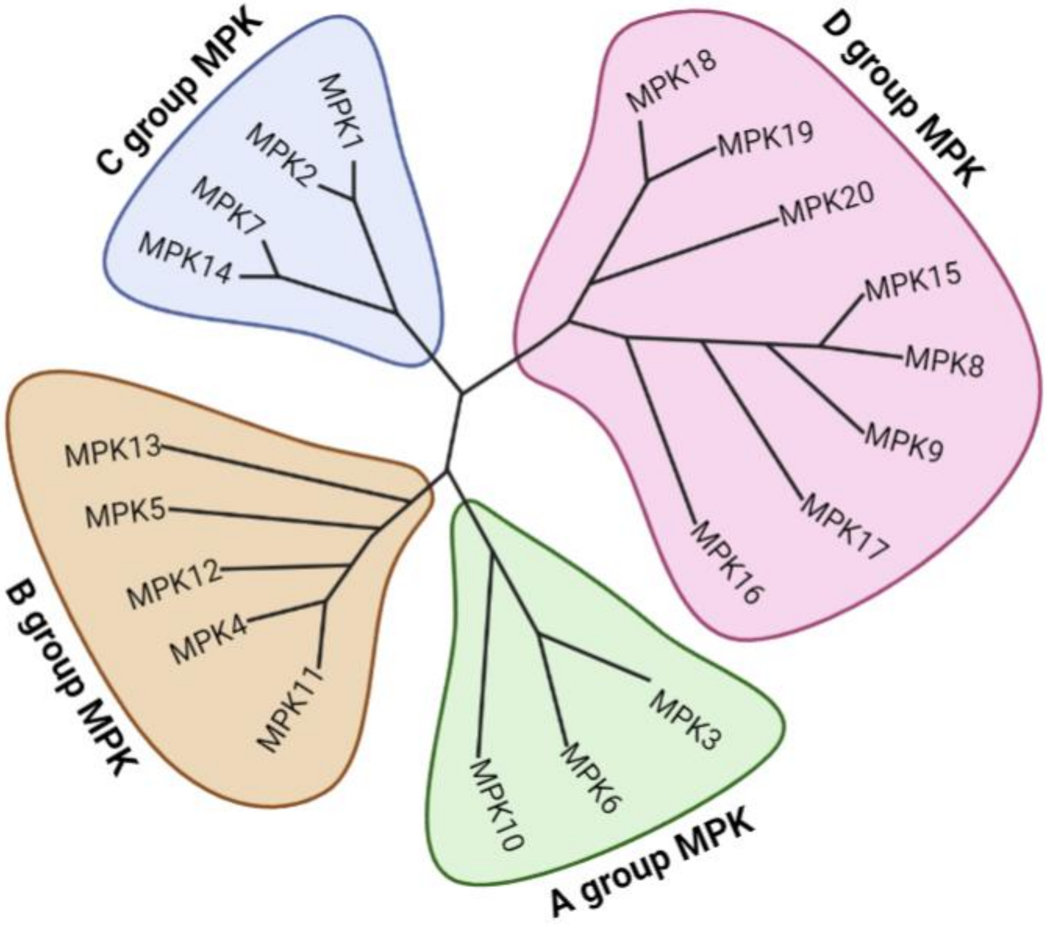

Supplemental Figure S1. Phylogenetic analysis of the 20 MPK proteins in *Arabidopsis thaliana*. Groups A, B, C and D are labeled with the corresponding MPKs present in each group. Sequence Viewer 8.0 was used to align the sequences and generate the phylogenetic tree.

A	MPK3	DFGLARPTS----ENDFMTEYVVTRWYRAPE
	MPK6	DFGLARVTS----ESDFMTEYVVTRWYRAPE
	MPK10	DFGLARATP----ESNLMTEYVVTRWYRAPE
B	MPK4	DFGLARTKS----ETDFMTEYVVTRWYRAPE
	MPK5	DFGLARTTS----ETEYMTEYVVTRWYRAPE
	MPK11	DFGLARTKS----ETDFMTEYVVTRWYRAPE
	MPK12	DFGLARTTS----DTDFMTEYVVTRWYRAPE
	MPK13	DFGLARTSN----ETEIMTEYVVTRWYRAPE
C	MPK1	DFGLARASNT---KGQFMTEYVVTRWYRAPE
	MPK2	DFGLARTSNT---KGQFMTEYVVTRWYRAPE
	MPK7	DFGLARTSQG---NEQFMTEYVVTRWYRAPE
	MPK14	DFGLART------YEQFMTEYVVTRWYRAPE
D	MPK8	DFGLARVSFNDAPTAIFWTDYVATRWYRAPE
	MPK9	DFGLARVSFNDAPSAIFWTDYVATRWYRAPE
	MPK15	DFGLARVSFNDAPTAIFWTDYVATRWYRAPE
	MPK16	DFGLARVAFNDTPTAIFWTDYVATRWYRAPE
	MPK17	DFGLARVSFTDSPSAVFWTDYVATRWYRAPE
	MPK18	DFGLARVAFNDTPTTVFWTDYVATRWYRAPE
	MPK19	DFGLARVSFNDTPTTVFWTDYVATRWYRAPE
	MPK20	DFGLARVAFNDTPTTIFWTDYVATRWYRAPE

Supplemental Figure S2: Alignment of Arabidopsis thaliana MPK activation loops. Protein kinase activation loops are defined by the sequences DFG and APE. In MPKs, activation is achieved by phosphorylation of a TXY motif (green). Arabidopsis MPKs form 4 phylogenetically distinct groups as indicated (A-D) with only the D-group have an aspartate (D) between the T and Y of the TXY motif, while groups A-C have a E in this position (*11*). The D-group enzymes also possess an insert in the loop that conforms to a protein phosphatase one (PP1) binding RVXF SLiM (blue).

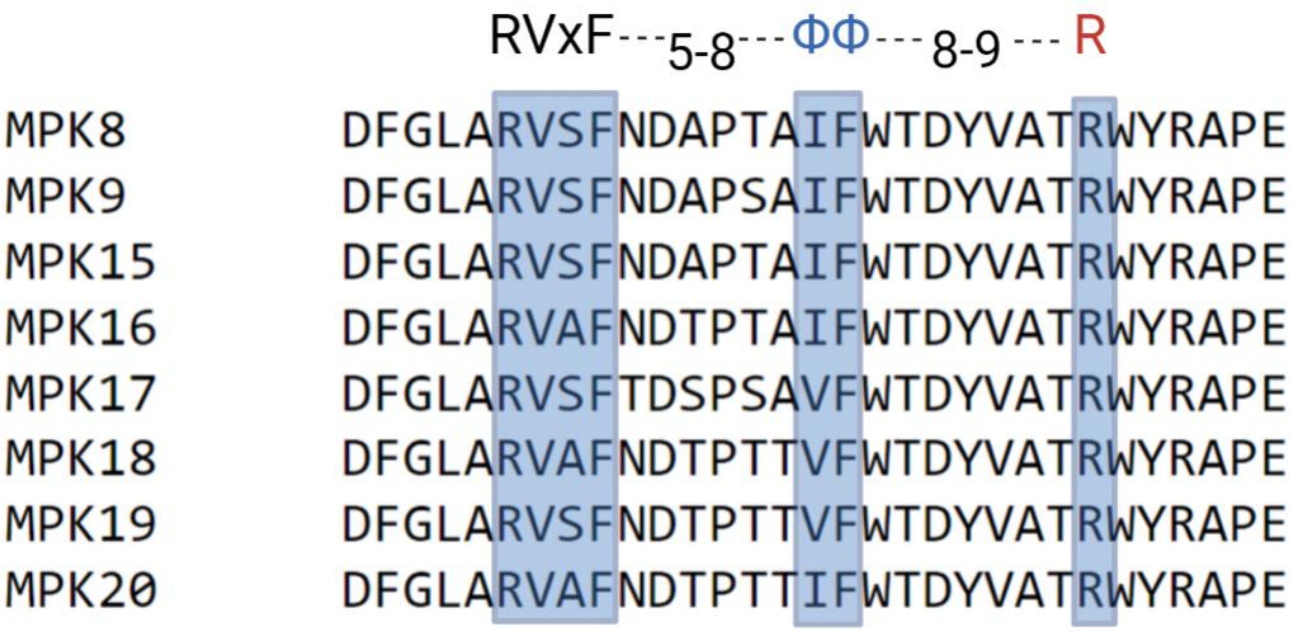

Supplemental Figure S3: *Arabidopsis thaliana* D group MPKs have an extended RVXF motif. The activation loops of the D group MPKs as defined by DFG and APE. Highlighted in blue are the amino acids associated with the extended RVXF motif, which starts with RVXF, then 5-8 amino acids C-terminal two hydrophobic residues (Val, Ile or Phe), and then 8-9 amino acids C-terminal an arginine (R). Each D-group enzyme conforms to this sequence motif further supporting the idea that these protein kinases recruit PP1.

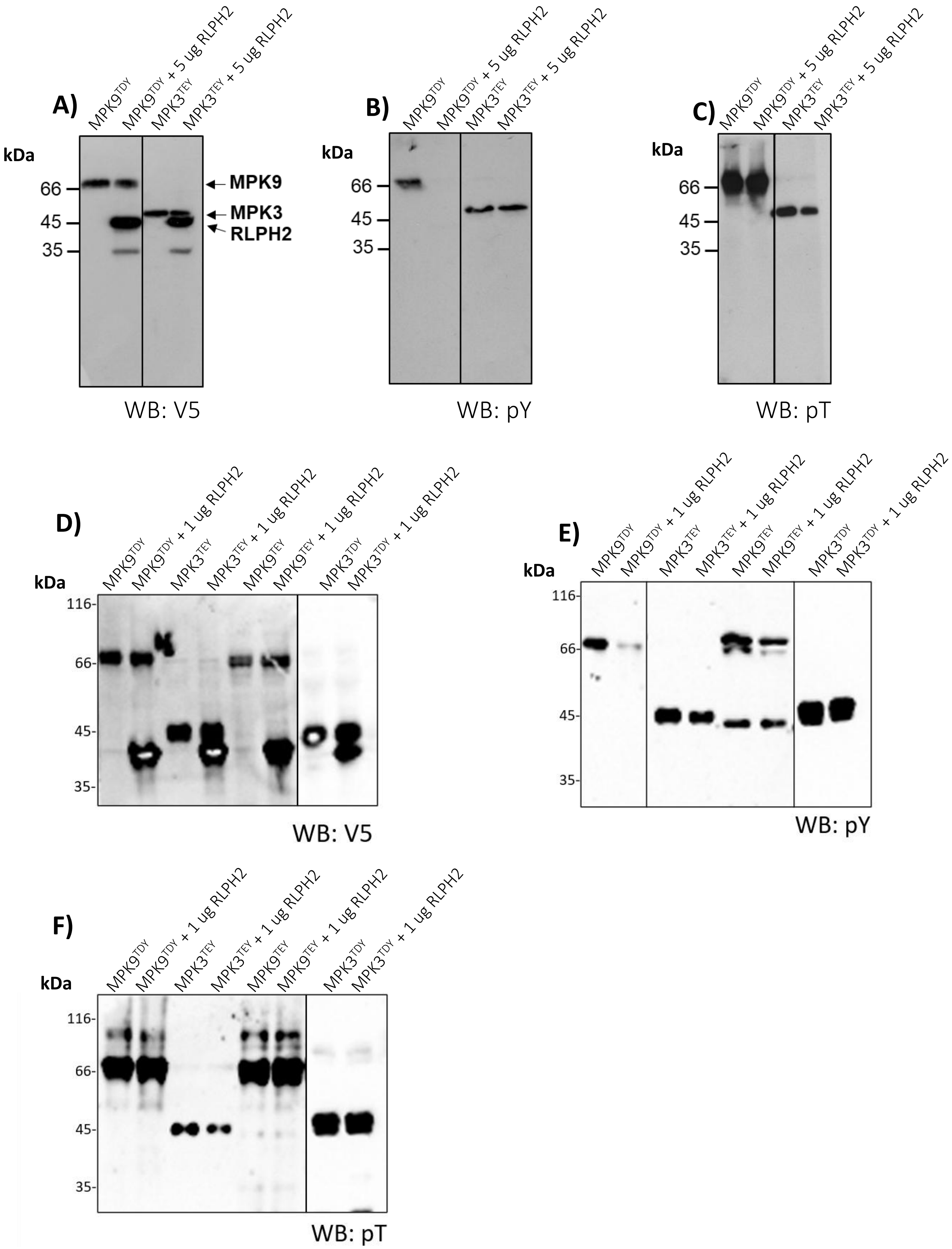

Figure S4. RLPH2 solely dephosphorylates pY of MPK9^{TDY}, but not MPK9^{TEY}, MPK3^{TEY} or MPK3^{TDY}. MPKs were dually phosphorylated in their activation loops (see methods) and treated with or without purified RLPH2. Western blotting was performed to assess the MPK phospho-status with either anti-pY or pT antibodies. A & D, anti-V5 immunoblot demonstrating equal loading. B, C, E & F, anti-phospho-tyrosine and anti-phospho-threonine immunoblots.

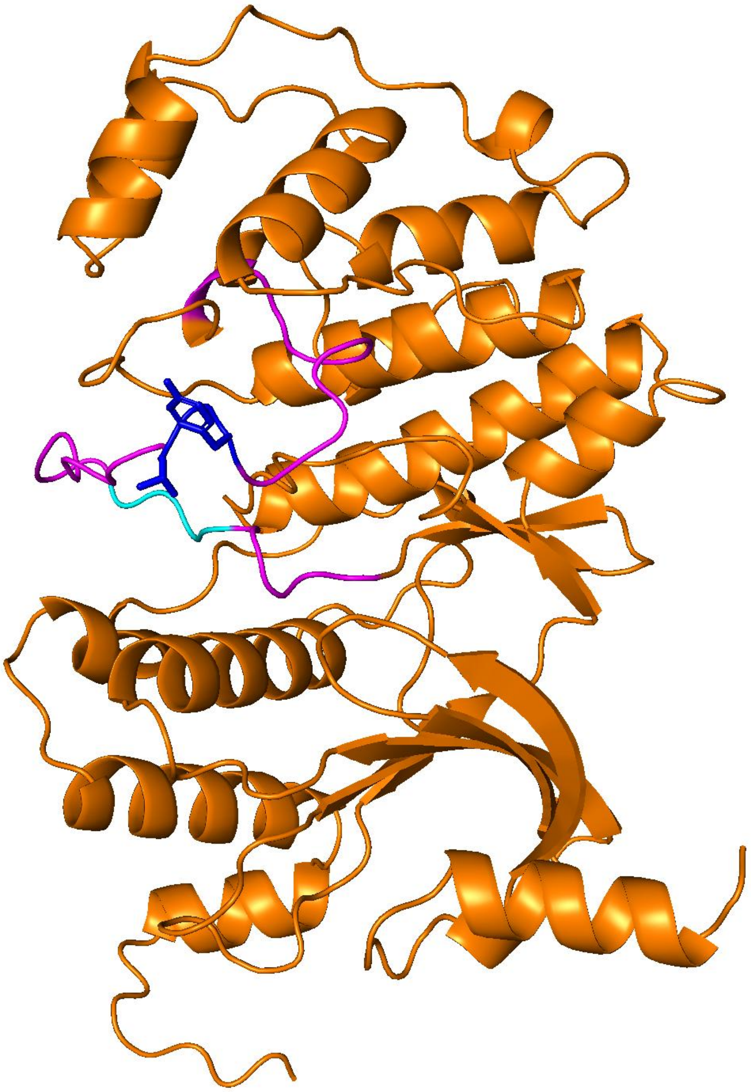

Figure S5. Predicted *Arabidopsis thaliana* MPK9 structure. The AlphaFold structure for MPK9 corresponding to UniProt ID Q9LV37 was downloaded from the AlphaFold Protein Structure Database (https://alphafold.ebi.ac.uk/). PyMol was used to visualize the structure and residues after tyrosine 376 were hidden for image clarity. Marked in pink is the activation loop, defined by DFG and APE, showing how the putative PP1 docking RVXF SLIM (light blue) is surface exposed. The activation loop TDY motif threonine (T) and tyrosine (Y) are presented with side chains shown (dark blue).

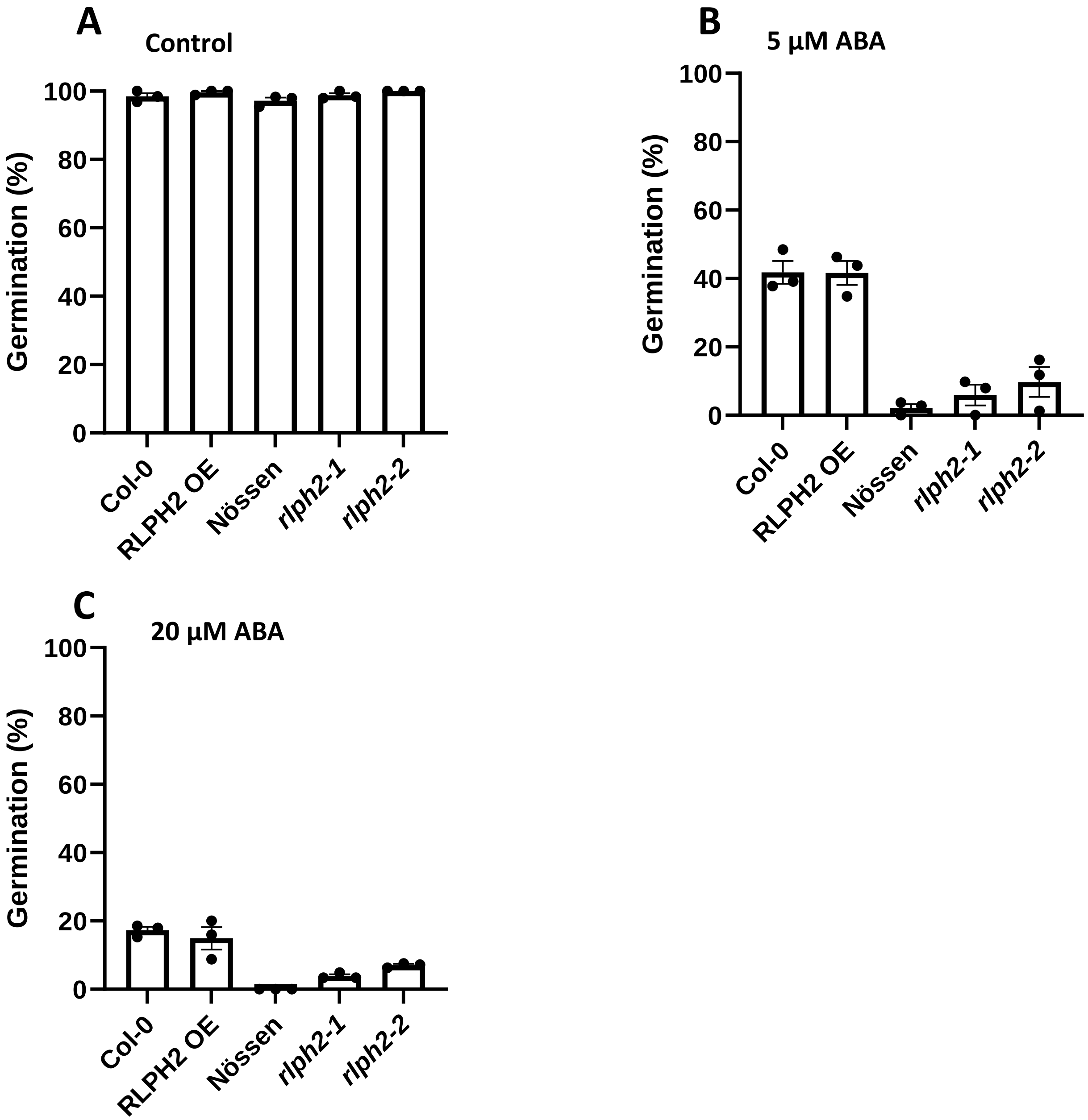

Supplemental Figure S6. Percentage germination after 7 days in the presence of ABA. ABA sensitivity of Nössen, *rlph2-1*, *rlph2-2*, Col-0 and 35S::RLPH2-Ctap line (*RLPH2-OE*). Seeds were stratified 4d at 4°C on MS agar medium without ABA (A, control) or in the presence of B, 5 and C, 20 µM ABA. The germination rate was scored after 7d on light at 21°C. Results represent germination means ± SE of three replicates of fifty seeds. Asterisks indicate statistical differences between Nössen and *rlph2* mutants or between Col-0 and the *RLPH2-OE* line, as determined by One-way ANOVA with post hoc Tukey's test ($P < 0.05$).

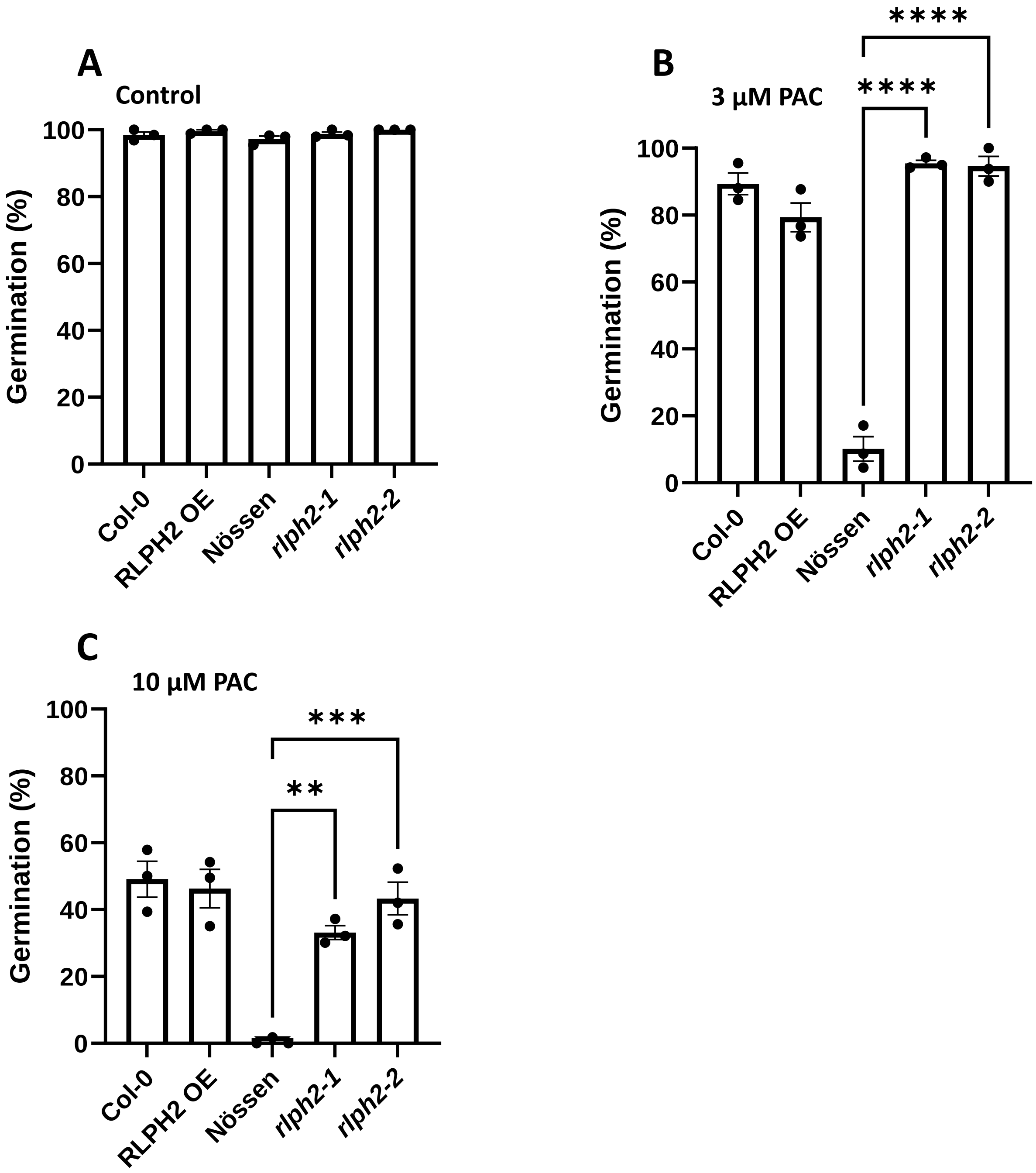

Supplemental Figure S7. Percentage germination in the presence of PAC after 7d. PAC sensitivity of Nössen, *rlph2-1*, *rlph2-2*, Col-0 and 35S::RLPH2-Ctap line (*RLPH2-OE*). Seeds were stratified 4d at 4°C on MS agar medium without PAC (A, control) or in the presence of B, 3, and C, 10 µM PAC. The germination rate was scored after 7d on light at 21°C. Results represent germination means ± SE of three replicates of fifty seeds. Asterisks indicate statistical differences between Nössen and *rlph2* mutants or between Col-0 and *RLPH2-OE* line, as determined by One-way ANOVA with post hoc Tukey's test ($P < 0.05$).

```
MPK8     DQLS--FMYPSGVDRFKRQFAHLEENQGKPGAAGGGRSTALHRHHASLPR
MPK15    NQLS--FMYPSGVDRFRRQFAHLEENQGP-----GGRSNALQRQHASLPR
MPK9     EQTS--FMYPSGVDRFKRQFAHLEENYGK-----GEKGSPLQRQHASLPR
MPK17    ENINSHFLYPSGVDQFKQEFARLEEHNDDEE---EHNSPPHQRKYTSLPR
MPK16    EPTN--FMYPSAVEHFKKQFAYLEEHYKNG----TSHNPPERQQHASLPR
MPK18    EGSN--FVYPSAIGHLRQQFTYLEENSSRN-----GPVIPLERKHASLPR
MPK19    EGSS--FLYPSAIGHLRKQFAYLEENSGKS-----GPVIPPDRKHASLPR
MPK20    DKAS--FLYPSAVDQFRRQFAHLEENSGKT-----GPVAPLERKHASLPR
```

Supplemental Figure S8: Conserved region of C-terminal tails of *Arabidopsis thaliana* D-group MPKs. The D-group MPKs were aligned and the conserved region among the C-terminal tails extracted and presented. The most conserved residues are highlighted in red.
