# Supplementary material for: Phospho‐Proteomics Identifies D‐Group MAP Kinases as Substrates of the Arabidopsis Tyrosine Phosphatase RLPH2
Source: Plant Direct. 2026 Jan 20;10(1):e70137. doi: 10.1002/pld3.70137 (PMC12817480; doi:10.1002/pld3.70137)
Supplement: Supplementary file 1 — Data S1: Peer review. [file PLD3-10-e70137-s003.docx]

Phospho-proteomics identifies D-group MAP kinases as substrates of the Arabidopsis tyrosine phosphatase RLPH2

Greg B Moorhead, Anne-Marie Labandera, Ryan Toth, Sierra Mitchell, Jayde J Johnson, Brooklyn McLaren Kurucz, Juliette Puyaubert, Emmanuel Baudouin, Richard Glen Uhrig.

Decisions Letter Round 1:

September 26, 2025

Prof. Greg B Moorhead

University of Calgary

Biological Sciences

Calgary T2N 1N 4

Canada

RE: Phospho-proteomics identifies D-group MAP kinases as substrates of the Arabidopsis tyrosine phosphatase RLPH2

Dear Dr. Moorhead:

Thank you for submitting to Plant Direct. All required reviews have been returned and we have now finished our evaluation of your manuscript. In light of the reviewers' and editor's comments, further revisions are needed before the paper can be accepted for publication in Plant Direct.

Please view the editors' and reviewers' comments below and use their suggestions as a guide while you work on your revision.

When uploading the revised version of this article, please be sure to include the following:

-A word document that contains your response to the reviewers. You should respond to each reviewer comment and note the changes made to the manuscript. If you do not agree with a reviewer's comment and choose not to make a suggested revision, please explain why. Please try to provide as complete an answer as possible to each reviewer's criticisms.

-A tracked changes document with each change highlighted

- A clean version of the latest version of the manuscript

To upload your revision, please click the link below.

https://plantdirect.msubmit.net/cgi-bin/main.plex?el=A6Lr3pZ2A2ux4I4A9ftdzDlpMXjEaGy0qfqWHqBiQZ

In order to provide as timely a service as possible, we ask that your revision is resubmitted within three months after receipt of this request. If an extension is needed, please send a request, along with a brief explanation, to the editorial office at plantdirect@wiley.com .

Please note that, in addition to publishing reviewer comments, the author's responses to review comments will also be published alongside the final version of the paper. If you would not like the author's responses to be published, please contact the editorial office at plantdirect@wiley.com .

Thank you very much for giving us an opportunity to review your work. I look forward to receiving the next version.

Sincerely,

Hongtao Ji

Gustavo MacIntosh

Editor, Plant Direct

---------------------------------------------------------------------------- Editor comments:

Two reviewers raised several questions. In my opinion, please address the comments from Reviewer 1 properly and try your best to respond to Reviewer 2's comments.

---------------------------------------------------------------------------- Reviewer comments:

Reviewer #1:

Major points:

1, The repeatability of all experiments and the number of independent repetitions are not described, making it impossible to judge the reliability of the results.

2, RLPH2-D-group MPK regulation in seeds, No evidence (e.g., seed-specific co-expression via in situ hybridization, pY levels of D-group MPKs in rlph2 seeds) directly connects RLPH2's biochemical activity to germination phenotypes;

Minor points:

3, In germination assay, the description of"freshly harvested seeds"lacks critical details (e.g., post-pollination harvest window, storage temperature/humidity), which influence seed dormancy and repeatability

4, Phosphorylation of MPK9 and MPK3 activation loops section

MPK9 (autophosphorylation) and MPK3 (CAMKK4-mediated phosphorylation) activation methods are described, but no data (e.g., pT/pY Western blot intensity)

5, Mutagenesis primers for MPK variants (e.g., MPK9RASA) are not provided,

6, Inconsistent terminology,"Protein phosphatase one" and "protein phosphatase 1" are used interchangeably for PP1; "rlph2" and "RIPH2" (typo) appear in Fig. S4 descriptions, creating confusion

7, Figure 4A, lack control for MPK9 and MPK9 variant to show equal amount of both proteins used in protein overlay assay

Reviewer #2:

The manuscript by Labendrea et al. on "Phospho-proteomics identifies D-group MAP kinases as substrates of the Arabidopsis tyrosine phosphatase RLPH2" is an interesting finding. The authors here identify that RLPH2 specifically phosphorylates Group D members of the Arabidopsis MAPK family. As out of Group A-D, only Group D members have the "TDY" motif at their activation loop, while the other three groups (Group A-) have the "TEY" motif. The authors very interestingly showed that RLPH2 specifically dephosphorylates MPK9, a group D MAPK, and NOT MPK3, a group A MAPK. The experiments are designed very inteeligently and results are more or less convincing. However, the manuscript has certain shortcomings that may require a bit more experimentation and clarification.

1. It will be interesting to show the protein-protein interaction of RLPH2 with more than one member of Group D MAPKS and compare the interaction with at least two members of Group A-C MAPKs. This could be Y2H assay, BiFC or what the authors think can be used in their setup.

2. Why does the germination pattern differ at 25 {degree sign}C for Atrlph2-1 and Atrlph2-2 mutants?

3. The experiments done with PAC and ABA application do not take the story further and seem half-cooked. Can authors add more experiments to bring the involvement of MAPKs in the observations, and that the dephosphorylation by RLPH2 actually plays a role?

4. Finally, it will be good to know why the dephosphorylation is reduced in "pTDpY" mptif of MPK9? Can the author throw some light by doing in silico docking experiments with a "DDD" or "EDE" motif?

Decision Letter Round 2:

December 23, 2025

Prof. Greg B Moorhead

University of Calgary

Biological Sciences

Calgary, N/A T2N 1N 4

Canada

MSID: 2025-01835-TWR1

MS TITLE: Phospho-proteomics identifies D-group MAP kinases as substrates of the Arabidopsis tyrosine phosphatase RLPH2

Dear Dr. Greg Moorhead:

I am pleased to inform you that your manuscript "Phospho-proteomics identifies D-group MAP kinases as substrates of the Arabidopsis tyrosine phosphatase RLPH2" has been accepted for publication in Plant Direct.

Your article will appear online in the next available issue of Plant Direct. To ensure your article gets published as quickly as possible, please pay attention to the steps detailed below. We have found that most of the delays happen at this stage, especially at the payment stage, so please respond as quickly as possible when prompted.

License Agreement: Once your article has been accepted it will move to Production and undergo admin and file checking - you may receive an email with any queries we have at this stage. When all required items are received by the publisher and queries resolved, the corresponding author will receive an email from Wiley's Author Services system which will ask them to log on at https://authorservices.wiley.com/bauthor and will present them with the appropriate license for completion. Your article cannot be published until both the signed license agreement and payment of the article fee have been received.

Payment of the Open Access Article Publication Fee: All articles published in Plant Direct are fully open access: immediately and freely available to read, download and share. Plant Direct charges a publication fee to cover the publication costs. The corresponding author for this manuscript should have already received a quote from the payments team (cs-openaccess@wiley.com) with the estimated article publication fee; please email cs-openaccess@wiley.com if this has not been received. The corresponding author should log on to the Wiley Author Services site, where the publication fee can be paid by credit card or an invoice. Pro Forma can also be requested. Payment of the publication charge must be received before the article will be published online.

Proofs: You will have the opportunity to look over your paper once more when you receive the author proofs for your article. The proofs will be with you in approximately two weeks. Please note that, in addition to publishing reviewer comments, the author's responses to review comments will also be published alongside the final version of the paper. If you would not like the author's responses to be published, please contact the editorial office at plantdirect@wiley.com .

Promotion of your article: You can help your research get the attention it deserves! Check out Wiley's free Promotion Guide for best-practice recommendations for promoting your work at www.wileyauthors.com/eeo/guide. And learn more about Wiley Editing Services which offers professional video, design, and writing services to create shareable video abstracts, infographics, conference posters, lay summaries, and research news stories for your research at www.wileyauthors.com/eeo/promotion.

Thank you again for your contribution to Plant Direct. If you have any questions, feel free to contact the editorial office at plantdirect@wiley.com .

Sincerely,

Hongtao Ji

Gustavo MacIntosh

Editor, Plant Direct

---------------------------------------------------------------------------- Editor comments

Thank you for your thoroughly efforts in addressing the concern raised.

---------------------------------------------------------------------------- Reviewer comments:

Reviewer #1:

The revised manuscript has satisfactorily addressed all the raised concerns. I recommend its acceptance for publication.

Reviewer #2:

The authors have substantially revised the manuscript and provided appropriate rebuttals where they have chosen to maintain their original claims. I have no further comments on the manuscript.

Response Letter Round 1:

Dear Editors,

Please find below our response to the reviewers’ comments as well as small changes to the

manuscript. Attached is also a new version of the manuscript with highlighted tracked

changes, a clean version of the manuscript and all the figures.

We thank you for your time.

Kind regards,

Greg B. Moorhead and Anne-Marie Labandera

-----------------------------------------------------------------------------------------------------------------------

Reviewer #1:

We want to thank you for all the suggestions and inquiries about our manuscript. We

have tried to answer as complete as possible all the questions.

Major points:

1, The repeatability of all experiments and the number of independent repetitions are not

described, making it impossible to judge the reliability of the results.

We apologize for not adding this earlier in some of the figures. The number of

replicates was added to the figure legends that didn’t have them. This was in Figure 3,

4 and S4.

2, RLPH2-D-group MPK regulation in seeds, No evidence (e.g., seed-specific coexpression

via in situ hybridization, pY levels of D-group MPKs in rlph2 seeds) directly connects

RLPH2's biochemical activity to germination phenotypes;

The reviewer is completely right. In this work we don’t show any direct evidence that

the germination phenotype observed in rlph2 mutants is indeed due to its activity

towards MPK8 or any D-group MPK in the seeds. Our knowledge on the function of the

members of the D-group MAPKs is very limited and sometimes overlaps with other

groups of MPKs, making it difficult to investigate phenotypes of RLPH2. Although the

connection of RLPH2 and MPK8 is not shown here, we came across the dormancy

phenotype in rlph2 knockout seeds after seeing that the D-group MAP Kinase MPK8

was shown to be important in the dormancy/germination process (24). We therefore

decided to start investigating if RLPH2 had a similar function. Future work, will include

a phosphoproteomics study in seeds instead of rosettes and a full characterization of

the phenotype observed.

In the manuscript we have added some clarification that we are not stating that there

is a direct link between RLPH2 and MPK8.

Minor points:

3, In germination assay, the description of "freshly harvested seeds" lacks critical details(e.g.,

post-pollination harvest window, storage temperature/humidity), which influence seed

dormancy and repeatability

We have added the information in the materials and methods.

4, Phosphorylation of MPK9 and MPK3 activation loops section MPK9 (autophosphorylation)

and MPK3 (CAMKK4-mediated phosphorylation) activation methods are described, but no

data (e.g., pT/pY Western blot intensity

Figure S4 shows the initial phosphorylation status of MPK9 and MPK3 in both TEY and

TDY versions in the lines where no RLPH2 is added. It was already shown that MPK9

autophosphorylates (16) and MPK3 is phosphorylated and activated by CAMKK4 (17)

Their phosphorylation status was checked with the pY and pT antibodies (Fig 3B and

S4). It is worth noting that these antibodies are not specific for the TxY motif but

general pY and pT antibodies.

5, Mutagenesis primers for MPK variants (e.g., MPK9RASA) are not provided

We have added a new supplemental table with the primers used for site directed

mutagenesis (supplemental table 3).

6, Inconsistent terminology, "Protein phosphatase one" and "protein phosphatase 1" are

used interchangeably for PP1; "rlph2" and "RIPH2" (typo) appear in Fig. S4 descriptions,

creating confusion

Thank you for pointing this out. We have checked the entire document to fix

inconsistencies in terminology.

7, Figure 4A, lack control for MPK9 and MPK9 variant to show equal amount of both proteins

used in protein overlay assay

We have added a new panel (panel A) in figure 4 to show the amount of both proteins

(MPK9RVSF

and MPK9RASA) used in panel B. This is an image that was generated when

the exact samples were prepped for the Figure 4 experiment to establish equal

loading. Moreover, an unspecific band appears in all samples, further supporting

equal loading (Fig 4B).

Reviewer #2

The manuscript by Labendrea et al. on "Phospho-proteomics identifies D-group MAP kinases

as substrates of the Arabidopsis tyrosine phosphatase RLPH2" is an interesting finding. The

authors here identify that RLPH2 specifically phosphorylates Group D members of the

Arabidopsis MAPK family. As out of Group A-D, only Group D members have the "TDY" motif

at their activation loop, while the other three groups (Group A-) have the "TEY" motif. The

authors very interestingly showed that RLPH2 specifically dephosphorylates MPK9, a group

D MAPK, and NOT MPK3, a group A MAPK. The experiments are designed very inteeligently

and results are more or less convincing. However, the manuscript has certain shortcomings

that may require a bit more experimentation and clarification.

We appreciate the positive overview and positive comments. We have tried to answer

as much as possible to all the questions. Please see below.

1. It will be interesting to show the protein-protein interaction of RLPH2 with more than one

member of Group D MAPKS and compare the interaction with at least two members of

Group A-C MAPKs. This could be Y2H assay, BiFC or what the authors think can be used in

their setup.

This would be a great addition in order to compare for example, specificities among

different Group D MAPKs. In this study, our aim was to validate biochemically the

results obtained by phosphoproteomics by comparing a representative member of

each group (D and A-C) and future work will involve interaction and substrate

specificity among MAPK groups as well as tissue specificity.

2. Why does the germination pattern differ at 25 {degree sign}C for Atrlph2-1 and Atrlph2-2

mutants?

We agree. It is intriguing that the pattern of germination is different among both rlph2

mutants, being rlph2-1 more dormant than rlph2-2. Although these lines have already

been confirmed as full knock outs in (9), we do not have an explanation for this

behavior. Since rlph2-1 germination rate is more similar to the wild type Nössen

seeds, we performed a statistical analysis between rlph2-1 and the wild type Nössen

line at 8 days to corroborate the significant difference between these two lines.

Indeed, pairwise t-test was significant (p < 0.0034). This has been added to the main

text.

3. The experiments done with PAC and ABA application do not take the story further and

seem half-cooked. Can authors add more experiments to bring the involvement of MAPKs in

the observations, and that the dephosphorylation by RLPH2 actually plays a role?

We agree that it would be interesting to further explore the link between RLPH2 and

MPK8 in the context of dormancy behavior. In our current work the direct connection

of RLPH2 and MPK8 is not shown. However, we explored and identified the dormancy

phenotype in rlph2 knock out seeds after being aware of the dormancy phenotype of

the D-group MAPK MPK8 (24). In future work we will include a phosphoproteomics

study of seeds instead of rosettes. We will explore further the role of RLPH2 in the

ABA-GA signaling/catabolism. We believe all this should be an independent future

study.

4. Finally, it will be good to know why the dephosphorylation is reduced in "pTDpY" motif of

MPK9? Can the author throw some light by doing in silico docking experiments with a"DDD"

or "EDE" motif?

We are unsure we fully understand this final question. Figure 3A shows phosphate

release in the process of dephosphorylation of the dually phosphorylated MPK3 and

MPK9 peptides by RLPH2. Here, we can see that MPK9 pTDpY gets preferentially

dephosphorylated. It is however intriguing why a single amino acid change from D to

E will generate such a difference in substrate specificity and for that we do not have

an answer. We know from our previous work, that RLPH2 prefers dual phosphorylated

TXY substrates (pTXpY) rather than single phosphorylated TXpY (8), however, until

now we did not know that the central amino acid of the TXY motif was also crucial to

increase specificity and dephosphorylation rates and unfortunately, we do not have an

explanation for this. As suggested, we did try in silico docking of the peptide

sequences for the activation loop of MPK9 as well as the full-length MPK9 sequence

into AlphaFold multimer with the RLPH2 sequence. In the “Peptides” version, the

peptides do dock at the active site interface. However, even for the dual

phosphorylated WT peptide it does not look accurate. The learning model is predicting

the pT is in the active site and not the pY. In the corresponding mutant peptides, they

do not face the active site for the D or E versions. Less can be said for the “Full

length" proteins as the RLPH2 protein as none of the expected MPK9 residues are

near the active site. Rather, the N-terminus of MPK9 is predicted to interact with the

active site of RLPH2.
